# Effects of computer-generated patterns with different temporal and spatial frequencies on choroidal thickness, retinal dopamine and candidate genes in chickens wearing lenses

**DOI:** 10.3389/fmed.2024.1469275

**Published:** 2024-12-10

**Authors:** Hong Liu, Frank Schaeffel, Marita Pauline Feldkaemper

**Affiliations:** ^1^Section of Neurobiology of the Eye, Ophthalmic Research Institute, University of Tübingen, Tübingen, Germany; ^2^Aier Institute of Optometry and Vision Science, Aier Eye Hospital Group, Changsha, China

**Keywords:** choroid, myopia, artificial visual stimuli, dopamine, EGR-1, chicken

## Abstract

**Purpose:**

Changes in choroidal thickness (ChT) are proposed to predict myopia development but evidence is mixed. We investigated time courses of choroidal responses, following different types of dynamic artificial stimulation in chicks with and without spectacle lenses, as well as changes in retinal dopamine metabolism and expression of candidate genes.

**Methods:**

Chicks were kept in an arena surrounded by computer monitors presenting dynamic checkerboard fields of small, medium and large size. Fields were displayed with different cycle frequencies, as ON (rapid rise, slow decay) or OFF (slow rise, rapid decay) temporal luminance profile. Refractive errors, ocular biometry and ChT were assessed. Dopamine metabolism and candidate gene expression levels were also measured. Stimuli were applied for (1) 3 h with no lens, (2) 3 h and monocular treatment with −7D or +7D lenses, (3) 3 or 7 days.

**Results:**

(1) The smallest fields caused the largest decrease in ChT. (2) Negative lens treatment induced on average 11.7 μm thinner choroids. ChT thinning was enhanced by 10 Hz-ON medium field size flicker which also reduced choroidal thickening with positive lenses. (3) With prolonged treatment, the choroid recovered from initial thinning in all groups although to varying degrees which were dependent on stimulus parameters. Relative ChT changes were positively correlated with the vitreal level of dopamine metabolites. Retinal *EGR-1* mRNA level was positively correlated with choroidal thickness. Retinal melanopsin mRNA was increased by 10 Hz-ON stimulation and choroidal *BMPR1A* mRNA increased with 10 Hz-OFF stimulation. On average, early choroidal thinning did not predict the amount of negative lens-induced eye growth changes after 7 days, whereas later ChT changes showed a weak association.

**Conclusion:**

Negative lenses caused long-lasting choroidal thinning, with some recovery during lens wear, especially after stimulation with 10 Hz. The dynamic stimuli modulated choroidal thinning but effects were small. There was little difference between ON and OFF stimulation, perhaps because the checkerboard patterns were too coarse. 10 Hz cycle frequency increased dopamine release. Less dopamine was correlated with thinner choroids. Result do not exclude a predictive value of choroidal thickening for future refractive development since we almost exclusively tested choroidal thinning effects.

## 1 Introduction

Wallman et al. (1) discovered in the early 90’s in chickens that the thickness of the choroid, the highly vascularized layer behind the retina, changes when the plane of focus in the retinal image is shifted. With a positive lens in front of the eye, the choroid thickens within hours, moving the retina closer to the focal plane. With a negative lens, the choroid thins, moving the retina closer to the focal plane. In the long term, defocus-induced choroidal thickness changes are followed by changes in growth of the outer coat of the eye, the sclera, leading to the compensation of lens-induced refractive errors (REs). Similar changes in choroidal thickness (ChT) were found in tree shrews, guinea pigs, marmosets and monkeys even though the effects on refractive state were smaller because the induced ChT changes were smaller and the eyes larger (2–4). A few studies in the chicken model have later addressed the question of whether ChT changes can predict ocular growth rates with all kinds of stimulation, with variable outcomes. While one study (5) showed that baseline ChT was neither related to baseline eye size, nor to subsequent eye growth rates, Nickla and Totonelly (6) measured an inverse correlation between ChT changes and axial eye growth rates in untreated chicken eyes. However, it was consistently found that baseline ChT did not predict the susceptibility to deprivation myopia (6, 7).

Recently, due to the advancements of optical coherence tomography (OCT) imaging, even tiny changes in ChT can be non-invasively measured in human subjects (8). It was found that (1) similar to chickens and monkeys, wearing a positive lens in one eye induced thickening of the choroid both in adults and children (9–11). (2) Studies in children also showed that developmental changes in ChT successfully predicted future myopia development (12). Furthermore, cross-sectional studies demonstrated that myopic children and myopic adults had thinner choroids than non-myopes. An inverse association between ChT changes and changes in eye growth was confirmed by Read et al. (13) who concluded that “Choroidal thickness exhibited an inverse association with the axial eye length changes, implying a potential role for the choroid in eye growth.” (3) Atropine, known to inhibit myopia development, also makes the choroid thicker and prevents choroidal thinning that is normally induced by wearing a negative lens (14).

In recent years, evidence has accumulated that visual stimuli other than defocus can also change ChT. However, it remained uncertain whether such changes can reliably predict whether a treatment can control myopia. Short term exposure (3 h) to computer-generated artificial stimuli on a screen that predominantly activate ON pathways caused choroidal thickening in chicks, while stimulation of the OFF pathways caused choroidal thinning (15). ChT returned to baseline after 7 days despite continued treatment (15). Unexpectedly, with both dynamic ON and OFF stimulation on a computer screen, with an underlying repetition frequency of 1 Hz, more myopia was induced by negative lenses than under continuous illumination with comparable brightness. Still, retinal dopamine release was higher with ON than OFF stimulation. The ChT changes measured after short-term exposure did not predict refractive states measured after 7 days. Possible explanations are that (1) the underlying repetition frequency (1 Hz) of the temporal sawtooth-shaped luminance profiles in each of the small fields of the checkerboard stimulus might have induced a myopic shift (16), (2) highly dynamic spatio-temporal pattern seen by the chickens made the retina more responsive to defocus and therefore made the negative lenses more effective, (3) in the long term, it does not matter whether stimulation is more ON or OFF because of adaptation of the ON and OFF pathways. Also, Nickla and Totonelly (6) found that the relationship between choroidal thickening and eye growth inhibition may be disrupted under certain experimental conditions. Given that researchers might rely on the assumption that thicker choroids predict myopia inhibition (1), there is an urgent need (1) to find out under which conditions these predictions remain valid and, (2) which mechanisms and biochemical pathways trigger such changes in ChT and, subsequently, scleral growth.

In the current study, we applied visual stimuli with different dynamic luminance profiles, provided on the computer screens as checkerboard patterns with random phases with respect to each other: (1) rapid ON with slow decay or (2) slow increase with rapid OFF or (3) temporal square wave luminance profiles. The repetition frequencies were either 0 Hz (stationary pattern), or 0.8 Hz, 1.2 Hz, 2.5 Hz, 6.5 Hz, 7.5 Hz, and 10 Hz. Individual fields of the checkerboards subtended either 1.7 deg. of visual angle (small squares, 28 × 28 pixels “SSQ”), 2.9 deg. (medium size squares, 48 × 48 pixels, “MSQ”), or 4.1 deg. (large squares, 68 × 68 pixels “LSQ”). Subsequent changes in ChT, eye growth, and myopia development were studied, including their time courses. To find out whether the presentation of a dynamic pattern itself, without ON or OFF dominance, may increase the sensitivity of the retina to defocus, square wave temporal luminance profiles were also tested.

To learn more about the messengers and biochemical pathways that control ChT and, subsequently, the growth of the sclera, some known markers of myopia development were studied. The time course of expression changes of established retinal and choroidal biomarkers is often unknown. We aimed to find out whether their expression level correlates with changes in eye growth and choroidal thickness after medium-term (3 days) and long-term (7 days) treatment. We focused on mRNA expression changes of the transcription factor early-growth response-1 (*EGR-1*) (17, 18), gap junction delta-2 (*GJD2*) (19), melanopsin (*OPN4*) (20) and neuropsin (*OPN5*) in the retina (21, 22). Choroidal markers shown to correlate with ChT, i.e., retinaldehyde dehydrogenase 2 (*RALDH2*) (23) and bone morphogenic protein receptor 1A (*BMPR1A*) (24) were also measured. In addition, we quantified the vitreal and retinal dopamine content and its metabolites, as they are known to be associated with changes in eye growth (25, 26).

## 2 Materials and methods

### 2.1 Animals and rearing conditions

White leghorn chicks were obtained from a local hatchery (Weiss, Kirchberg, Germany) at postnatal day 1. They were raised in a temperature and humidity-controlled animal facility. Water and food were supplied *ad libitum*. The light cycle was 11:13 light/dark (8:30 AM to 6:30 PM) with an illuminance of approximately 500 lux during the light phase. All experiments were conducted in accordance with the statement of the Association for Research in Vision and Ophthalmology (ARVO) for the use of Animals in Ophthalmic and Vision Research. Procedures were approved by the commission for Animal Welfare of the Medical Faculty of the University of Tübingen. Water and food were supplied *ad libitum* under all experimental conditions.

### 2.2 Visual stimulation: ON-, OFF- and square wave stimuli

Dynamic ON or OFF or square wave stimuli were developed using Visual C++ 8.0 as previously described (15). Stimuli consisted of a checkerboard pattern in which the fields had a repetitive sawtooth-shaped temporal luminance profile, with ON stimuli generated by a rapid rise in brightness and slow linear decay and OFF with a slow rise and rapid decay. Square wave stimuli with either bright or dark fields with both rapid ON and OFF and 0.5 duty cycle were also used. Cycles in all fields were randomly phase shifted with respect to each other, using the rnd() function in C++. Cycle frequency could be adjusted by selecting the number of pixel brightness steps in the temporal brightness slopes of each field.

For stimulation, chickens were kept in a perspex container (“arena” 60 × 60 cm) as previously described (15). Four computer screens (Acer KG271A, 61 cm; Acer, New Taipei City, Taiwan; resolution 1,920 × 1,080 (3.15 px/mm); refresh frequency 144 Hz), displaying the stimuli, were placed behind the transparent walls. In addition, the stimuli were projected from above on the white cardboard covering the floor by two video projectors (Acer, P1383W, resolution 1,280 × 800, contrast 13,000:1). The backlights of the computer screens were white LEDs and the projectors contained mercury high-pressure lamps. The combined spectrum of the computer screen and projector lamps perceived by the chicks in the arena was continuous, ranging from 380 to 780 nm (Supplementary Figure S1). Average illuminance in the arena was 400 lux. Chicks were kept in the arena during the experiment and housed in the animal facility before and after exposure. Chicks could freely move in the “arena” with water and food supplied in small bowls *ad libitum*.

### 2.3 Experiments

#### 2.3.1 Effects of short-term dynamic ON or OFF stimulation with different temporal and spatial frequencies on ChT in chickens with normal visual experience

Seven chickens per group were exposed to ON and OFF stimuli for 3 h, from 10:00 AM to 1:00 PM. The duration of 3 h was chosen based on a previous set of experiments that showed significant changes in choroidal thickness after 3 h (15). ChT was determined by spectral domain optical coherence tomography (SD-OCT) before and after the exposure period. The numbers of squared fields in the checkerboard pattern on the screen were 68 × 39 (28 × 28 pixels = 8.9 × 8.9 mm, small squares, “SSQ”), 40 × 22 (48 × 48 pixels = 15.2 × 15.2 mm, medium size squares, “MSQ”) or 28 × 16 (68 × 68 pixels = 21.5 × 21.5 mm; large squares, “LSQ”). Naturally, the visual angles of each field varied considerably with the viewing distances of the chickens. If they were in the center of the arena, the viewing distance was about 30 cm, and the visual angles were 1.7 deg. (SSQ), 2.9 deg. (MSQ) and 4.1 deg. (LSQ), equivalent to spatial frequencies of about 0.3, 0.17 and 0.12 cyc/deg.

Cycle frequencies of 0 Hz, 0.8 Hz, 1.2 Hz, 2.5 Hz, 6.5 Hz, 7.5 Hz, and 10 Hz were tested.

#### 2.3.2 Effects of short-term dynamic ON or OFF stimulation with different temporal and spatial frequencies on ChT in chickens treated with lenses

Chicks were treated monocularly with a −7D lens or a +7D lens. The same experimental set-up as described under (2.3.1) was used.

#### 2.3.3 Effects of long-term exposure (7 days) and medium-term exposure (3 days) to ON-, OFF and square wave stimuli on ChT and eye growth

The medium square size (MSQ) of the checkerboard pattern was used. Baseline measurements of refractive error (RE), ocular biometry, and ChT were taken at 8–10 days of age. A −7D lens was placed in front of one eye of the animals in the morning of the following day, the contralateral eye served as internal control. Stimuli were presented daily between 9:00 AM and 6:00 PM, and they spent the remaining time of the day in the dark. After a treatment period of 3 days or 7 days measurements of refraction, biometry, and ChT were taken, and retinal and choroidal samples were collected for high pressure liquid chromatography (HPLC) and quantitative RT-PCR (qRT-PCT) analyses.

##### 2.3.3.1 7 days treatment

Chicks were exposed to checkerboard stimuli (medium square size, MSQ) at different frequencies (1.2 Hz, 10 Hz, or static) and different wave types (ON, OFF, or square). Two different control groups were used: chicks in the room light (RL) control group (“RL-7d,” *n* = 7) were raised under white room light (400 lux, spectral range, spectral range of light 380 to 780 nm), while chicks in the second control group (“static-7d,” *n* = 6) were placed in an “arena” displaying stationary brightness in the checkerboard patterns. Illuminances for both control groups were approximately 400 lux, as in all other experimental groups. In addition, chicks were randomly assigned to the 1.2-Hz ON stimulus group (1.2-ON-7d, *n* = 7), 1.2-Hz OFF stimulus group (1.2-OFF-7d, *n* = 7), 1.2-Hz square wave stimulus group (1.2-square-7d, *n* = 7), 10-Hz ON stimulus group (10-ON-7d, *n* = 7), 10-Hz OFF stimulus group (10-OFF-7d, *n* = 7) and 10-Hz square wave stimulus group (10-square-7d, *n* = 7).

##### 2.3.3.2 3 days treatment

Since we did not find a significant effect of the 1.2 Hz stimulus on ChT in the 7 day treatment groups, we omitted the 1.2 Hz ON and OFF group. Chicks were monocularly treated with a −7D lens and randomly assigned to one of the following groups: room light group (RL-3d, *n* = 7), static pattern group (static-3d, *n* = 7), 10-Hz ON stimulus group (10-ON-3d, *n* = 7), 10-Hz OFF stimulus group (10-OFF-3d, *n* = 7) and 10-Hz square wave stimulus group (10-square-3d, *n* = 6).

### 2.4 Measurements

#### 2.4.1 Refractive error and ocular biometry

Refraction and ocular biometry were measured before and after treatment for the medium and long-term treatment. An automated version of infrared photoretinoscopy was used to measure the refractive error (27). Five readings per eye were taken and averaged for analysis. Ocular dimensions were measured using A-scan ultrasonography with a 11 MHz probe as previously described (28). The speed of sound in the lens of the chick was previously determined by Wallman and Adams (29). The cornea was topically anesthetized with one drop of 2% xylocaine solution before measuring. The depth of the anterior chamber (ACD), lens thickness (LT), vitreous chamber depth (VCD) and axial length (AL) were recorded, with five repeated measurements.

#### 2.4.2 Spectral domain optical coherence tomography

OCT represents a highly precise, fast and convenient technique to measure ChT in alert chickens (28). OCT measurements (Spectralis OCT, Heidelberg Engineering, Germany, resolution mode: high speed, scan angle: 30 degrees, scan type: B-scan, 768 × 496 pixels, line scan, eye tracking not engaged, scan rate of the live image 8.8 frames/s, wavelength of measurement 1,060 nm) were taken once per day during 10:00 to 10:30 a.m., as previously described (28). The −7D lens was removed before measurement and cleaned carefully. The measurements were taken in a short time (usually within 1 min), and the lens was immediately put back on after each measurement. To ensure consistent measurements in the same fundal area, we held alert chicks by hand in front of the OCT camera and manually adjusted the position of their heads until the cornea was aligned perpendicular to the optical axis of the OCT camera and the fundal layers could be seen on the screen. Optimal alignment of the chicken eye was assumed when the image of the pupil in the left screen window was centered and the scan of the fundal layers in the right window was horizontally aligned. Scans became tilted when the eyes were not properly aligned. Repeated measurements involved re-alignment of the chick head and the eye in each case. Five images were analyzed for each eye and ChT was measured at 5 positions in each image. The approximate lateral distance between each of the five measurement positions within each image was 80 μm, covering a total lateral distance of about 320 μm. The ChT was measured manually using the publicly available software ImageJ,^1^ being determined as the distance between the retinal pigment epithelium layer and the outer boundary of the choroid [as described in Liu et al. (30)].

#### 2.4.3 Sample preparation

The chicks were sacrificed by inhaling an overdose of ether and the eyes were immediately enucleated. The eyeballs were cut perpendicularly into halves with a razor blade, 1 mm posterior to the ora serrata. The anterior segment was discarded, leaving only the posterior eye cup. The vitreous body was then removed and quickly frozen in liquid nitrogen. A circular tissue sample with an 8-mm diameter was cut from the central posterior eye cup using a biopsy punch, as previously described (31). The biopsy samples were consistently taken from the circular region just above the root of the pecten, in the central area. The sample was then transferred to a Petri dish under a dissecting microscope, with the retina facing up. Typically, the retina detached easily and was peeled off with an ophthalmic hook. The pigment epithelium was discarded, and the choroid was separated from the sclera using forceps and a hook. Any small clusters of retinal pigment epithelial cells remaining on the choroid were carefully brushed away under visual control. The retina was separated into two halves, one for high pressure liquid chromatography (HPLC) and one for qRT-PCR analysis, while the choroid was only used for HPLC analysis. Tissues were immediately frozen in liquid nitrogen and stored at −80°C for subsequent analysis.

#### 2.4.4 Measurement of dopamine and metabolites via high pressure liquid chromatography

All vitreal samples were weighed and homogenized in 750 μL mobile phase (Thermo Fisher Scientific, Sunnyvale, CA, United States) using a tissue lyser and 5-mm stainless steel beads (TissureLyser LT, Qiagen, Hilden, Germany) at 50 Hz for 4 min. For retinal samples, 350 μL of mobile phase was added instead. A volume of 50 μL of retinal samples was reserved for protein concentration determination (BCA Protein kit, Thermo Scientific, Rockford, IL, United States). Following homogenization, all samples were centrifuged at 14,000 g for 10 min at 4°C. The resulting supernatant was filtered using a 0.2 μm nylon membrane filter (Thermo Scientific, Rockwood, MI, United States), and 25 μL of the filtered sample were directly injected into the HPLC system. Samples were analyzed for catecholamine and indolamine content via HPLC (Ultimate 3000 LC with electrochemical detection ECD 3000RS, Thermo Fischer Scientific) with coulometric detection utilizing an established HPLC method in our lab (32). In brief, a hypersil C18 column was used (150 mm × 3 mm, 3 μm) together with a test mobile phase (Thermo Fischer Scientific) containing 10% acetonitrile and 1% phosphate buffer. The flow rate was 0.4 mL/min and the potential at the first and second electrode was set to +370 and −200 mV, respectively. Dopamine, 3,4-dihydroxyphenylacetic acid (DOPAC), homovanillic acid (HVA, a metabolite of dopamine), serotonin and 5’-Hydroxyindolylessigsäure (HIAA) concentrations were determined with a high reproducibility (98%). The biogenic amine content in the retina was quantified as nanograms per milligram of protein (ng/mg protein), while in the vitreal, the concentration of the substances was determined relative to the wet weight (ng/100 mg wet weight). As described by others, vitreal DOPAC levels can be used as a sensitive measure of DA release from the retina (33). In addition, vitreal HVA levels can be used as an indirect measure of dopaminergic activity, as it has been shown that vitreal HVA levels correlate significantly with vitreal DOPAC levels (32).

#### 2.4.5 Real-time PCR analysis

Total RNA was extracted from retina and choroid samples using the Qiagen RNeasy Mini Kit (Qiagen, Hilden, Germany), and the resulting RNA was treated with DNase Inactivation Reagent (AM1906, Invitrogen, Waltham, Massachusetts, United States) to eliminate contaminating chromosomal DNA according to the manufacturer’s instructions. The concentration of RNA (ng/μL) was determined using the NanoDrop^®^ ND-1000 Spectrophotometer (Thermo Fisher Scientific, Wilmington, United States), and the purity and quality of the nucleic acid were assessed via the OD260/OD280 nm absorption ratio. Subsequently, 1 μg of retinal RNA or 500 ng of choroidal RNA was reverse transcribed to generate first-strand cDNA using a mixture of oligo(dT)15 and random primers. GoScript (Promega, Madison, United States) was used to reverse transcribe the RNA while the addition of a Rnase inhibitor (Thermo Fisher Scientific, Wilmington, United States) prevented RNA degradation.

The primer pair for melanopsin (*OPN4*) (Table 1) was designed using the Primer-BLAST tool (NCBI) (forward primer 5′-TAGGCGTCTGGCTGTACTCT-3′; reverse primer 5′-TGTGTAGGCACGGACTGATG-3′; product length 136 Bp). The primer pairs for amplification of *GJD2*, *OPN5*, *RALDH2* and *BMPR1A*, *β*-actin *(ACTB)* and Hypoxanthinephosphoribosyl-transferase (HPRT) have already been published [*RALDH2* and *EGR-1* (34); *GJD2* (35); *BMPR1A* (36); *OPN5* (21); *BACT* and *HPRT* (37), Table 1].

**Table 1:** Primer sequences.

Gene	Primer sequence	
	Forward primer (5′-3′)	Reverse primer (5′-3′)
*HPRT*	TGGCGATGATGAACAAGGT	GCTACAATGTGGTGTCCTCCC
*ACTB*	CTGAACCCCAAAGCCAAC	CACCATCACCAGAGTCCATCAC
*OPN4*	TAGGCGTCTGGCTGTACTCT	TGTGTAGGCACGGACTGATG
*RALDH2*	GCATCTGCTGCCTTCTCCC	AGGCGAGCTGCTCTCACTG
*EGR-1*	CTTGACCACGCACATCCGC	GCTGAGACCGAAGCTGCCT
*GJD2*	TTGGTGTTCATGTTTGCTGTCA	CCAGCCCAAGTGGTTCAGTT
*BMPR1A*	TGTCACAGGAGGTATTGTTGAAGAG	AAGATGGATCATTTGGCACCAT
*OPN5*	GGGCTGGCTTCTTCTTTGGCTGTGG	CAGGCAGATAAAGGCATGGTGT

Efficiency of the primers was calculated with the equation *E* = 10^(−1/slope)^, where the slope of the standard curve was derived from serial cDNA dilutions. PCR was performed using the an iCycler device (CFX96TM System, BioRad, Hercules, United States), with the cycling conditions set as follows: pre-activation phase for 3 min at 95°C, followed by 39 cycles of 95°C for 10 s, 59°C for 15 s, and 72°C for 15 s, with a final extension at 95°C for 3 min. Melting curve analysis was conducted subsequently. Single pure products were verified with a single peak. PCR was carried out in a 96-well plate with 2 ng of cDNA per well, using the QuantiNova SYBR Green PCR Kit (Qiagen, Hilden, Germany). Triplicate reactions were performed for all samples. Hypoxanthinephosphoribosyl-transferase (*HPRT*) and β-actin (*ACTB*) were used as reference genes. The mRNA expression level was analyzed using mean normalized expression (MNE), which is directly proportional to the amount of the target gene relative to the reference gene. MNE was calculated separately for either *ACTB* or *HPRT* using the mean cycle threshold (CT) value of the target and reference genes and taking the efficiency (*E*) of the PCR reaction into account:

$$MNE={\left(E_{\mathrm{reference}}\right)}^{\mathrm{CTreference},\mathrm{mean}}/{\left(E_{\mathrm{target}}\right)}^{\mathrm{CTtarget},\mathrm{mean}}$$

#### 2.4.6 Statistics

Data are shown as the mean ± SEM. The changes in axial length, vitreous chamber depth and ChT were also calculated (ΔX, ΔN, lens- treated eye = X; fellow eye = N). The difference in the changes in treated and contralateral control eyes is referred to as the relative change (ΔX − ΔN). A power calculation was undertaken to determine the group sizes required to achieve 80% power in observing a 1 D change in refractive development (when the standard deviation (SD) is approximately 0.6 D), a 0.075 mm change in axial length growth (when SD is 0.045 mm), 0.25 ng difference in retinal dopamine content (when SD is 0.15 ng) and 50 μm ChT change (when SD is 30 μm). With a group size of 7 chicks, differences between treatment groups should become significant at the 5% level. The normal distribution of the variables was confirmed via Shapiro–Wilk normality test.

The interocular differences (lens-treated vs. fellow eye) were analyzed with paired *t*-test. The difference between each two groups (exp. vs. exp., fellow vs. fellow) were compared using two-way mixed analysis of variance (ANOVA), with “eye” and “group” being within- and between-subject factors, respectively. Tukey’s multiple comparisons test was applied in *post hoc* analyses. Multifactorial ANOVAs were used to evaluate the impact of image size, character, frequency and lens treatment on ChT changes and the interaction effect of these variables. If a significant main effect of one of the parameters was detected, the data was split and one-way ANOVA was used for comparison, followed by Tukey’s multiple comparisons test. ChT measured at different times after stimulation was compared with the respective individual thickness at baseline using a two-way ANOVA with repeated measures and Dunnett’s test for multiple comparisons. “time” and “eye” were considered as two within-subject factors. Values of *p* < 0.05 were considered significant. Statistical analyses were done using commercial software JMP 16 (SAS Institute, Cary, NC, United States) and GraphPad prism 8 (San Diego, CA, United States).

## 3 Results

### 3.1 Effects of short-term (3 h) dynamic ON/OFF stimulation with different temporal and spatial frequency on changes in ChT

#### 3.1.1 Effects of ON/OFF stimulation in the control group with normal vision

**Figure 1:**
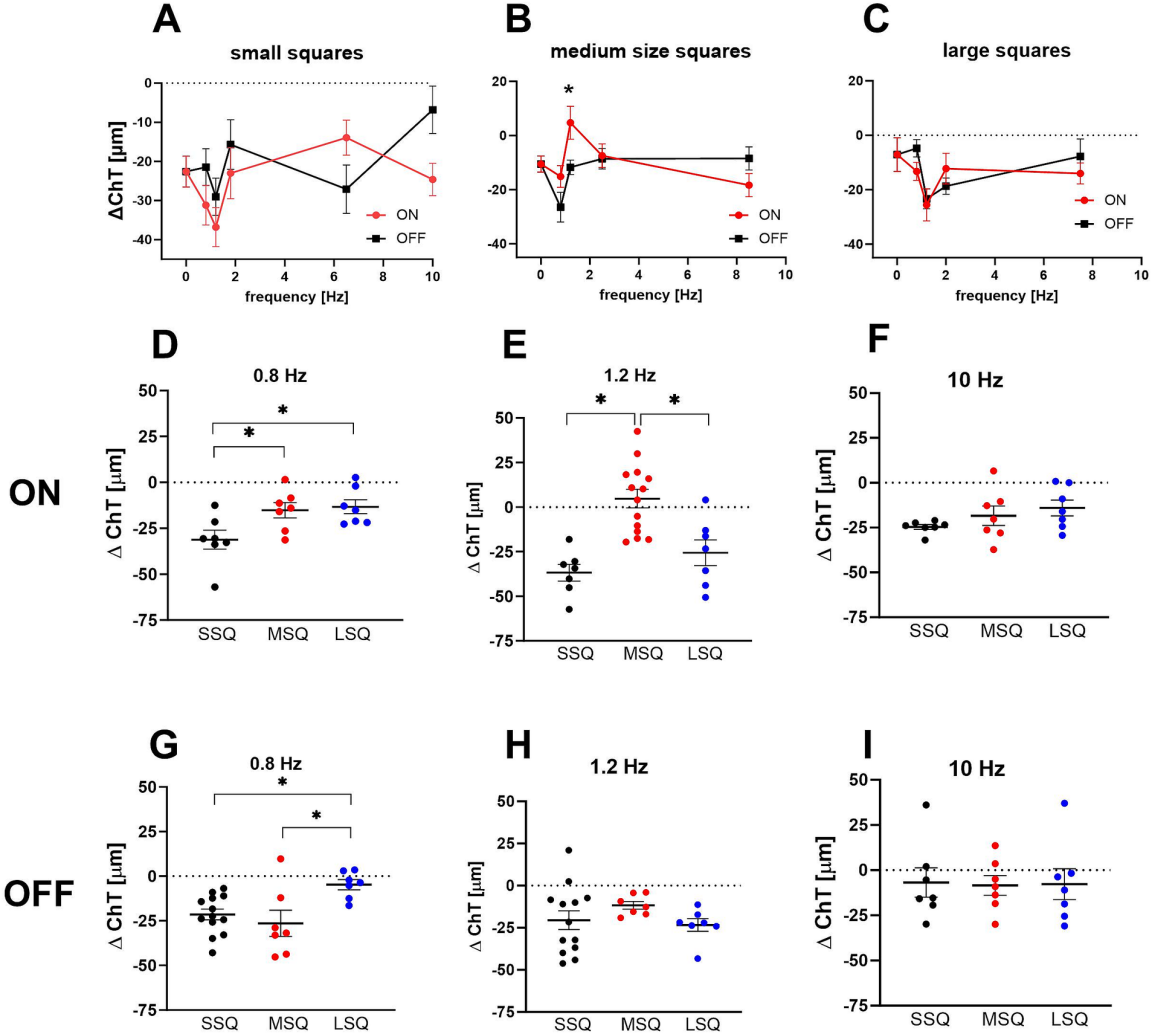
**(A–C)** Effect of short-term dynamic ON/OFF stimuli with different temporal and spatial frequency on changes in choroidal thickness in control chicks. The size of the stimuli had a significant main effect, with the smallest squares causing the largest decrease in ChT. Only one of the stimuli (B: 1.2 Hz ON, MSQ) induced an increase in choroidal thickness (+4.8 ± 6.5 μm) while OFF stimuli at this field size and frequency caused a significantly thinner choroid (−11.7 ± 2.7 μm). Graphs show mean data ± SEM. Un-paired *t*-tests: ^*^p < 0.05. **(D–I)** The interaction between temporal and spatial frequency on choroidal thickness changes was highly significant. In combination with 0.8 Hz flicker large stimuli (LSQ) produced significantly smaller changes in choroidal thickness than small stimuli (SSQ) **(C,D)**, whereas under 1.2 Hz flicker choroidal thickness was thickest in the chicks treated with medium size ON stimuli **(E)**. Graphs show mean data ± SEM. One-way ANOVA, Tukey *post hoc* analysis: ^*^p < 0.05.

The field sizes of the checkerboard pattern had a significant main effect on short-term ChT changes with the smallest squares (SSQ) causing the largest decrease in ChT (Multifactorial ANOVA: size: *p* < 0.0002; mean delta ChT: SSQ: −22.8 ± 2.3 μm, MSQ: −11.2 ± 2.2 μm, LSQ: −13.4 ± 1.9 μm) while frequency had no significant effect (*p* = 0.2877) and there was no difference between ON and OFF stimulation (*p* = 0.421). There was a significant interaction between temporal frequency and size (Multifactorial ANOVA: frequency × size: *p* = 0.0024). The data was therefore split according to stimulus size (Figures 1A–C) and frequency (Figures 1D–I). It was found that the sawtooth-shaped temporal luminance profile (ON/OFF) had a significant effect when small squares were used (Figure 1A; *p* = 0.0024; mean ChT change ON: −25.9 μm, OFF: −19.0 μm) and frequency significantly influenced ChT when medium size squares were used (Figure 1B, *p* = 0.010). Only one stimulus (1.2 Hz ON, MSQ) induced choroidal thickening (+4.8 ± 6.5 μm), all others made the choroid thinner. For this reason, medium size square (MSQ) stimuli were used for the medium- and long-term treatment.

Interactions between spatial and temporal frequencies are shown in Figures 1D–I. At 0.8 Hz, the largest checkerboard stimuli (LSQ) caused less choroidal thinning than the smallest (SSQ) with both types, ON and OFF (one-way ANOVA, Figure 1D ON: *p* < 0.01; Figure 1G OFF: *p* = 0.0189). The medium checkerboard stimulus (MSQ) had a similar effect on ChT changes as the large stimulus (LSQ) in the ON condition, and the small (SSQ) stimulus in the OFF condition. At 1.2 Hz the medium size squares (MSQ) induced significantly less choroidal thinning than LSQ and SSQ, and only with the ON stimulus (Figures 1E,H). There was no effect of square size on ChT changes at 10 Hz temporal flicker stimulation (Figures 1F,I).

#### 3.1.2 Effects of short-term exposure in chicken with negative lenses (small size checkerboard patterns) and of medium size checkerboard patterns on eyes with negative or positive lenses

**Figure 2:**
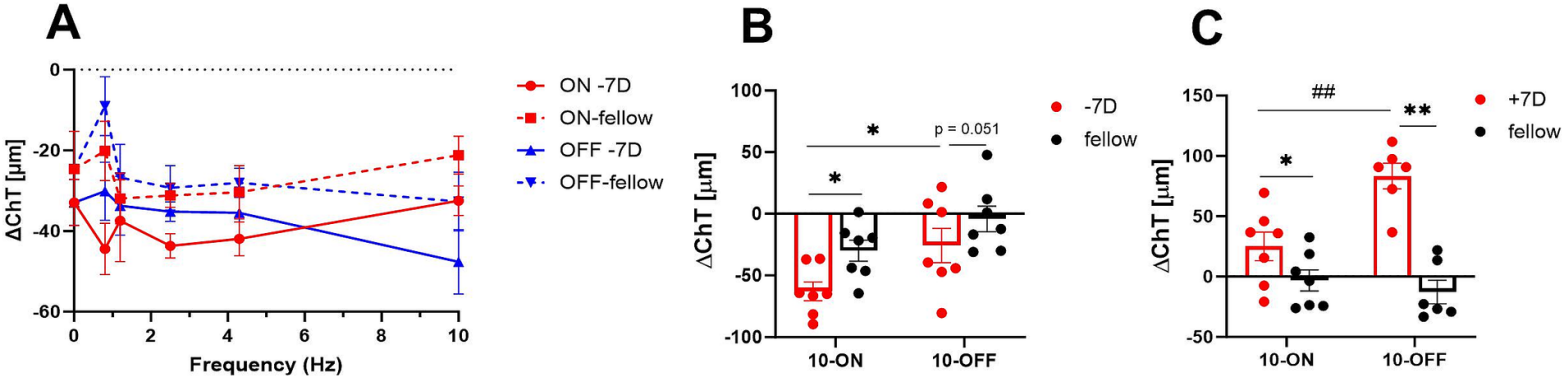
**(A)** Effect of negative lens treatment on choroidal thickness changes under different flicker frequencies (small square size). Three hours of treatment induced on average 11.7 μm more choroidal thinning compared to the contralateral eyes. Also, the flicker frequency of the checkerboard pattern has a significant main effect. The graph shows mean data ± SEM. **(B,C)** Comparison of the effect of plus lens treatment and negative lens treatment on choroidal thickness. Three hours of negative lens treatment induced a significantly larger reduction in choroidal thickness under ON flicker of 10 Hz and medium size checker-board stimuli compared to 10 Hz OFF flicker (10 Hz ON vs. 10 Hz OFF: −62.8 ± 7.6 μm vs. −25.6 ± 13.9 μm), whereas 3 hours of plus lens treatment induced a larger increase under OFF flicker of 10 Hz (trend, *p* = 0.052). Graphs show mean data ± SEM. Paired *t*-test: ^*^p < 0.05. Unpaired *t*-test: ^##^*p* < 0.01.

Chicks were treated unilaterally with −7D lenses, the contralateral eye was left open and served as control. The small size checkerboard-pattern was used (SSQ). Three hours of negative lens treatment induced on average 11.7 μm thinner choroids compared to the control eyes (Figure 2A). The treatment (negative lens vs. control) and frequency had a significant main effect (multifactorial ANOVA: treatment: *p* < 0.0001; frequency: *p* = 0.037) but the type of flicker (ON/OFF/no flicker) had not (Multifactorial ANOVA: type of flicker: *p* = 0.20).

Medium size checkerboard pattern (MSQ) was used in addition. Three hours of negative lens treatment induced significantly more choroidal thinning with ON flicker, compared to OFF flicker (Figure 2B: 10 Hz ON vs. 10 Hz OFF: −62.8 ± 7.6 μm vs. −25.6 ± 13.9 μm), whereas three hours of positive lens treatment induced a larger increase with ON flicker (Figure 2C: 10 Hz ON vs. 10 Hz OFF: −83.3 ± 10.6 vs. −25.1 ± 12.8 μm).

### 3.2 Effects of medium- and long-term treatment with ON, OFF and square wave stimuli (medium size, MSQ) on myopia development and eye growth

#### 3.2.1 Refraction

**Figure 3:**
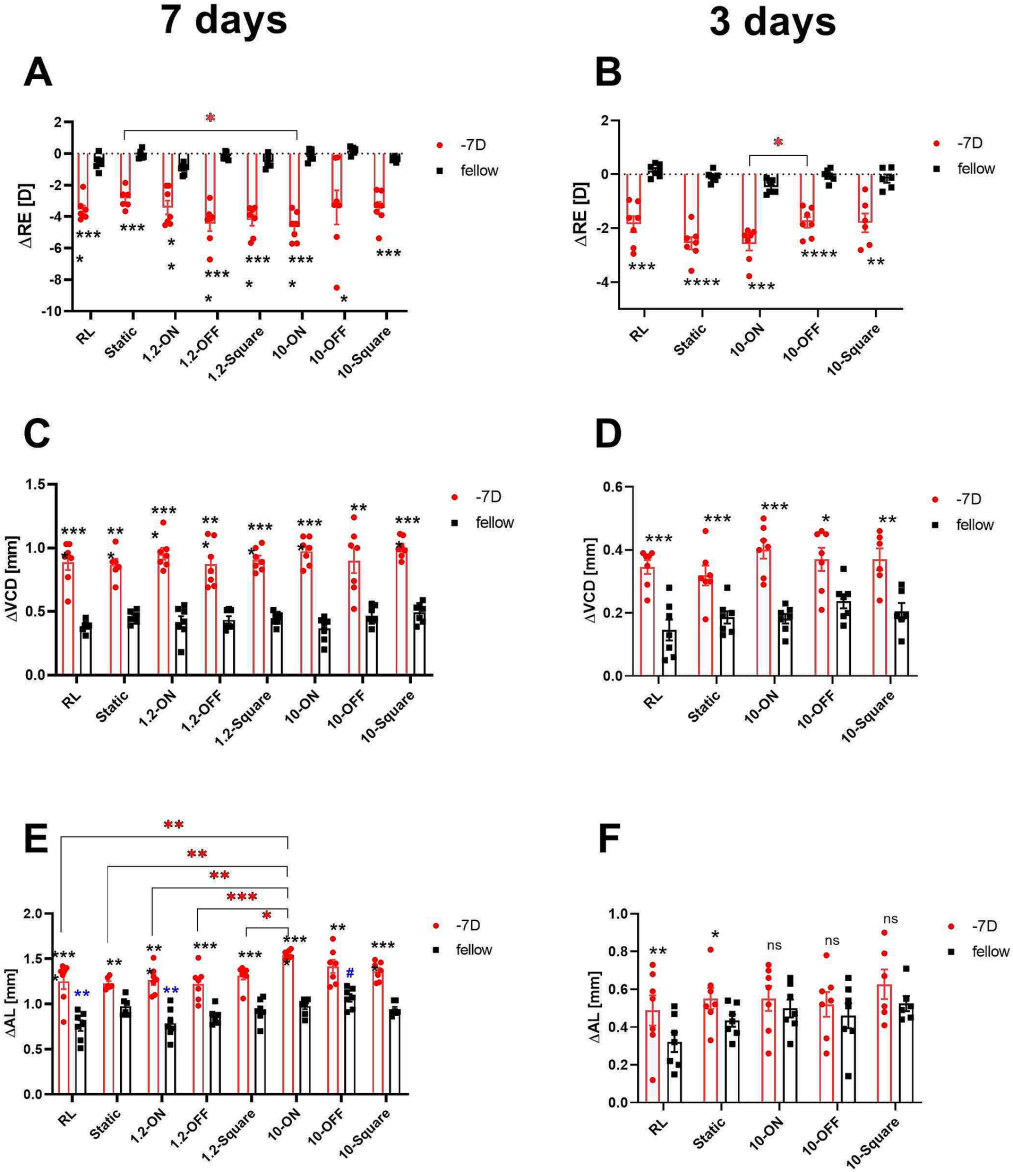
Effects of different stimuli on eye growth after treatment for 7 days (A,C,E) and for 3 days (B,D,F). After 7 days of treatment, negative lens-treated eye exposed to 10-Hz ON stimulus were more myopic than those treated with a static pattern **(A)**. The 10 Hz ON stimulus also induced more myopia than the 10-Hz OFF stimulus in the medium-term experiment **(B)**. None of the stimuli influenced the increase in VCD induced by negative lens **(C,D)**. On the contrary, in the long-term experiment, a significant effect on the change in AL in LIM eyes was observed in the 10-ON group, which exhibited longer AL compared to the RL, static, and 1.2-Hz groups **(E)**. In addition, the AL of the fellow eyes in 10-OFF group was longer compared to the RL group. No significant differences were found among the lens treated groups after 3 days of treatment. However, it is worth noting that also no interocular differences between the contralateral control eyes and the −7D lens treated eyes were found in all groups exposed to 10-Hz flicker (ON, OFF, static) **(F)**. Data are shown as mean ± SEM. The black asterisks denote interocular differences assessed via paired *t*-tests. Red asterisks indicate differences among groups determined by Tukey’s *post hoc* test. Blue asterisks indicate differences compared with the 10-OFF group (denoted by blue pound symbols). ^*^p < 0.05, ^**^p < 0.01, ^***^p < 0.001, and ^****^p < 0.0001; ns: not significant.

All groups of chicks exhibited a significant myopic shift after 3 or 7 days of lens wear. There was no significant difference in refractive development in the fellow eyes. The negative lens-treated eyes were more myopic after 7 days of treatment under 10 Hz dynamic ON flicker (10-ON) than those under a stationary stimulus, as shown in Figure 3A (∆RE: −4.66 ± 0.34 D vs. −2.86 ± 0.26 D, *p* = 0.03). Furthermore, 3 days of treatment with 10 Hz ON stimulation induced more myopia than 10 Hz OFF stimulation in the lens treated eye (∆RE: 10-ON vs. 10-OFF: −2.59 ± 0.24 D vs. −1.78 ± 0.20 D, *p* = 0.04, Figure 3B).

#### 3.2.2 Ocular biometry

After 3 and 7 days of treatment, VCD was significantly longer in negative lens-treated eyes, compared to the contralateral eyes in all groups (Figures 3C,D). Compared to the control group in room light, none of the stimuli had a significant effect on the growth of the vitreous chamber depth, neither in the lens-treated eyes nor in the contralateral control eyes (Figures 3C,D).

As expected, the 7-day treatment induced a significantly greater eye length growth in the negative lens-treated eyes compared to the respective untreated control eyes, but not to the same extent in all groups. A comparison of the treatment groups showed that the change in axial length in the negative lens-treated eyes was significantly greater in the 10-ON group than those in the RL, static, and 1.2-Hz groups (∆AL: 1.55 ± 0.02 mm in 10-ON vs. 1.25 ± 0.09 mm in RL, *p* = 0.002; vs. 1.23 ± 0.03 mm in static, *p* = 0.001; vs. 1.27 ± 0.06 mm in 1.2-ON, *p* = 0.004, vs. 1.22 ± 0.07 mm in 1.2-OFF, *p* = 0.0005; vs. 1.31 ± 0.04 mm in 1.2-square, *p* = 0.03, Figure 3E). Furthermore, the fellow eyes in the 10-OFF group developed significantly longer eyes compared to RL and 1.2-ON groups (∆AL: 1.07 ± 0.04 mm in 10-OFF vs. 0.76 ± 0.06 mm in RL, *p* = 0.001; vs. 0.79 ± 0.06 mm in 1.2-ON, *p* = 0.004, Figure 3E).

After 3 days of treatment, the lens-treated eyes were longer than the fellow eyes only in the RL and static stimulus groups. All groups kept under a 10 Hz stimulus, whether ON, OFF, or square, did not have significantly longer axial lengths in the negative lens-treated eyes compared to their fellow eyes. There was no significant difference in axial length between the different groups after only 3 days (Figure 3F).

### 3.3 Effect of frequency and stimulus pattern on ChT changes

**Figure 4:**
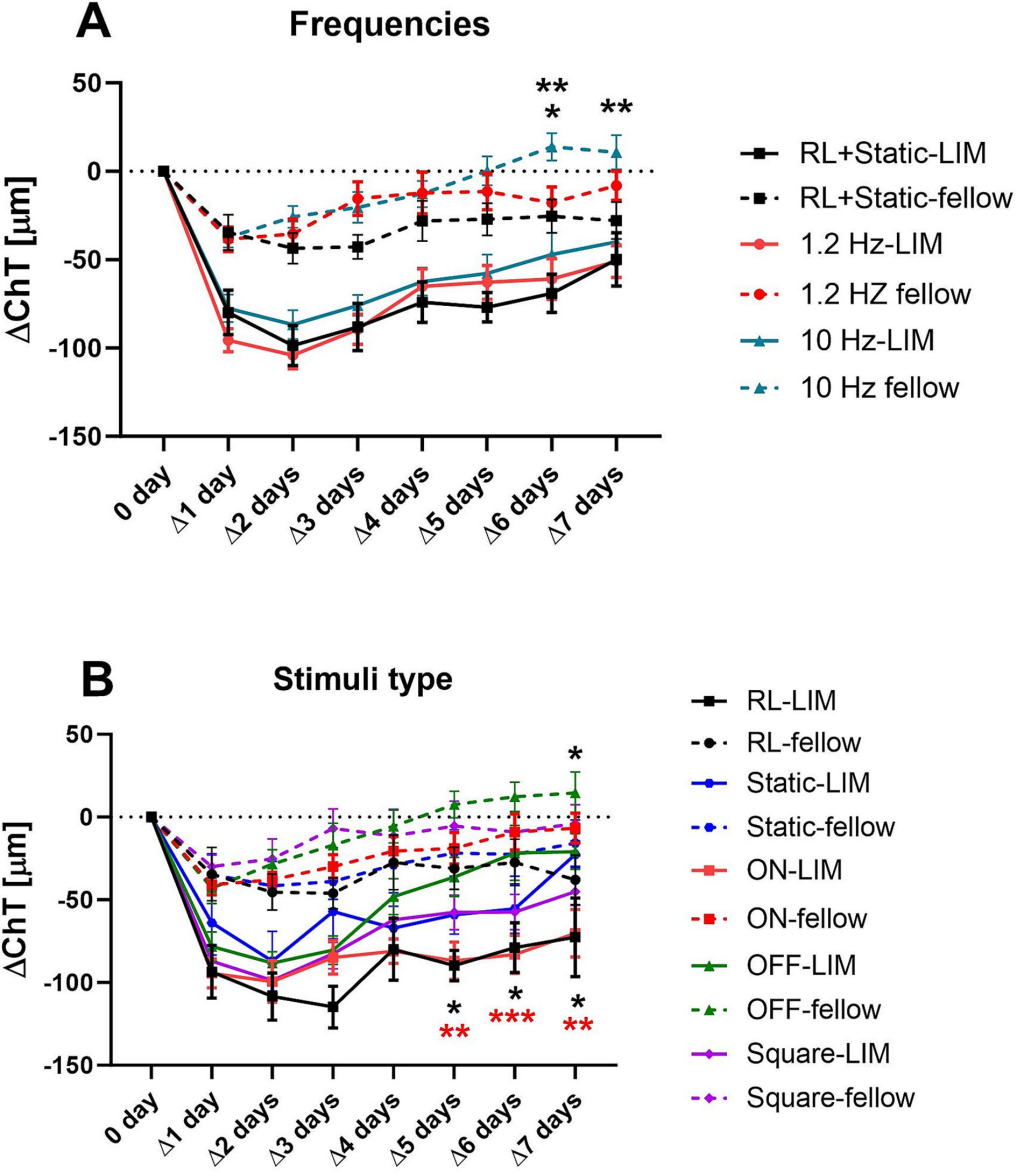
Effects of different frequencies **(A)** and wave types **(B)** on the change of ChT (compared to the respective baseline measurement). All stimulus paradigms resulted in initial choroidal thinning, with some recovery over the 7 days. The high frequency stimulation resulted in an increase in ChT in the 10 Hz fellow eyes on the 6th and 7th day **(A)**. When analyzing the effect of different stimulus patterns, only OFF stimuli increased the ChT, in the fellow and lens treated eyes **(B)**. The asterisks in **(A)** indicate the level of significance in comparison to the 10-Hz group, while those in **(B)** indicate the level of significance in comparison to the OFF group. Data were shown as mean ± SEM. ^*^p < 0.05, ^**^p < 0.01, and ^***^p < 0.001.

We combined the treatment groups belonging to the same category (same frequency or same stimulus pattern, respectively) to analyze the main effect of flicker frequency on ChT changes on the one hand and stimulus type on the other hand. All stimulus paradigms resulted in initial choroidal thinning, with some recovery over the 7 days (also in room light). Our results showed that the time course of ChT changes was not affected by the frequency of flicker light in the lens treated eyes. But in the fellow eyes, we detected a significant increase in ChT as shown in Figure 4A. Here, the choroid was significantly thicker after 6 days of treatment with 10-Hz flicker (∆ChT: 13.84 ± 7.83 μm) compared to the fellow eyes in the experiment with 1.2 Hz flicker (−17.73 ± 8.76 μm, *p* = 0.02) and also, if compared to fellow eyes in room light or with static patterns (−25.34 ± 9.47 μm, *p* = 0.005). On day 7, the choroid was significantly thicker in fellow eyes at 10 Hz flicker (∆ChT: 10.79 ± 9.64 μm), compared to room light or with static patterns (−27.84 ± 10.41 μm, *p* = 0.007). At 1.2 Hz, the difference to fellow eyes was no longer significant (−8.14 ± 8.27 μm, *p* = 0.24, Figure 4A).

When analyzing the different patterns of stimuli (ON, OFF, square wave, static), we found that, compared to room light, only the OFF stimulus had a significant effect on ChT in both negative lens-treated and fellow eyes. The choroid remained thicker in eyes treated with the OFF stimulus and negative lenses, compared to room light or compared to ON stimulation, at least during the last 3 days of the experiment (∆ChT on the 5th day: −36.26 ± 11.90 μm in the OFF group vs. −89.76 ± 9.20 μm in the RL group, *p* = 0.04, and vs. −86.89 ± 11.25 μm in the ON group, *p* = 0.009; ∆ChT on the 6th day: −21.68 ± 16.55 μm in the OFF group vs. −78.91 ± 15.05 μm in the RL group, *p* = 0.02, and vs. −83.11 ± 11.53 μm in the ON group, *p* = 0.0007; ∆ChT on the 7th day: −20.85 ± 10.79 μm in the OFF group vs. −72.66 ± 23.67 μm in the RL group, *p* = 0.049, and vs. −70.24 ± 14.35 μm in the ON group, *p* = 0.01, respectively, Figure 4B). Furthermore, in fellow eyes, the choroid was also thicker in eyes treated with the OFF stimulus, compared to in room light on the 7th day (14.60 ± 12.65 μm vs. −37.96 ± 14.89 μm, *p* = 0.046, Figure 4B). No significant changes were detected when compared to ON, square or static pattern stimuli.

### 3.4 Effects of different stimuli and time on ChT

**Figure 5:**
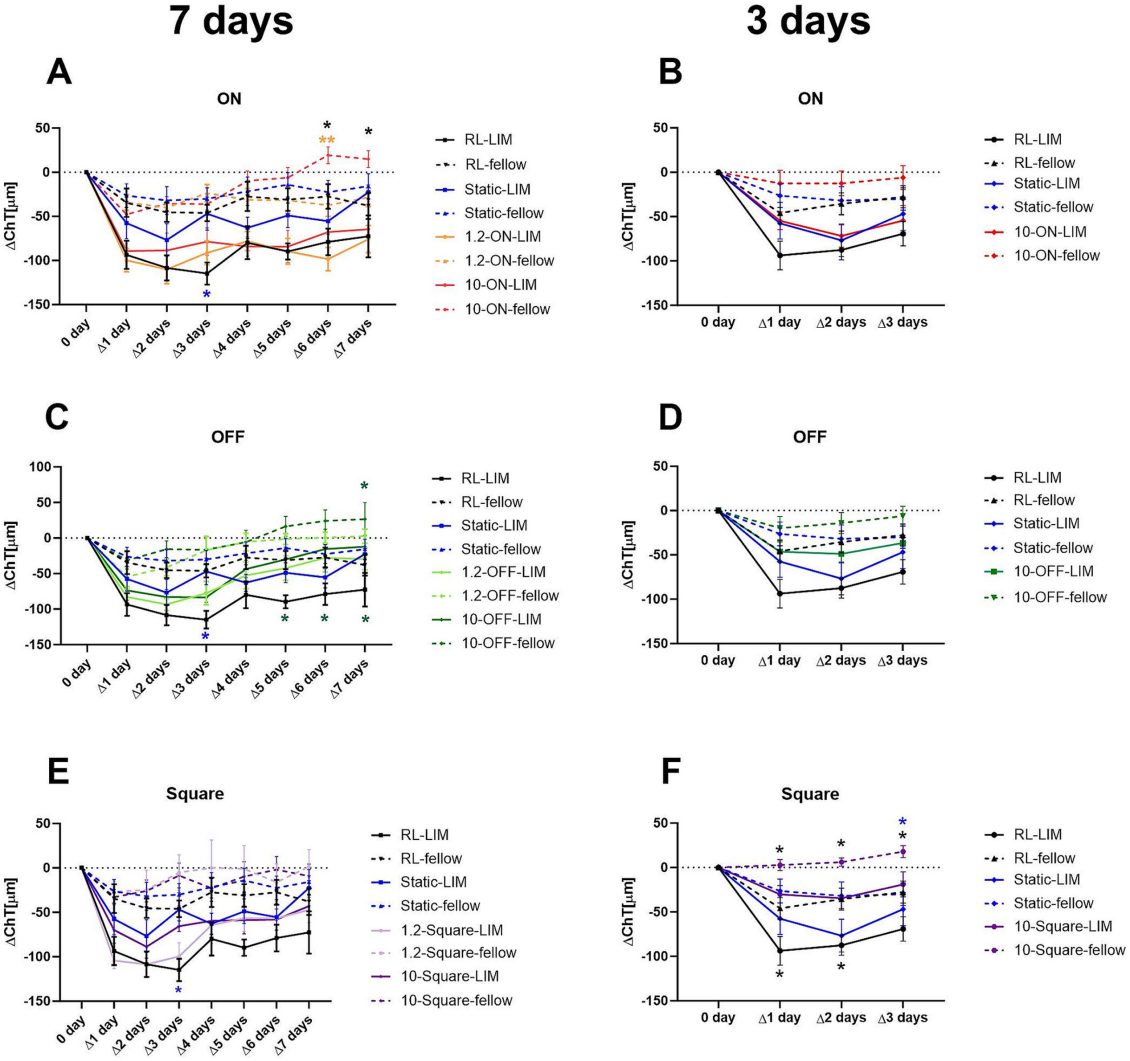
Effect of different stimuli on the change of ChT after 7 days **(A,C,E)** and medium-3 days of treatment **(B,D,F)**. In the long-term experiment, the 10-Hz ON stimulus thickened the choroid in the fellow eyes **(A)**, whilst the 10-Hz OFF stimulus increased the ChT in both eyes **(B)**. The square wave stimulus did not have a significant influence on the ChT **(C)**. In the medium-term experiment, no significant differences were detected with ON or OFF stimuli **(B,D)**. However, the choroid was thickened by the square wave stimulus in both eyes **(F)**. Data were shown as mean ± SEM. ^*^p < 0.05 and ^**^p < 0.01.

ChT decreased strongly during the first day of stimulation. This was true for all eyes, including the fellow eyes, but here to a lesser extent. The 10 Hz OFF stimulus had the strongest effect on the ChT. In this group, the choroid was significantly thicker from day 5 to the end of the experiment than in chicks kept under room light (RL vs. 10 Hz-OFF: ∆5 days: −89.76 ± 9.20 μm vs. −30.14 ± 17.40 μm, *p* = 0.048; ∆6 days: −78.91 ± 15.05 μm vs. −15.47 ± 27.81 μm, *p* = 0.03; ∆7 days: −72.66 ± 23.67 μm vs. −12.20 ± 15.82 μm, *p* = 0.045, two-way repeated measures ANOVA with Tukey’s test, Figure 5C). A similar effect was observed in the 10 Hz-OFF fellow eyes, although only on the last day. In the 10 Hz ON fellow eyes, ChT was also increased at days 6 and 7 (∆6 days: 19.37 ± 9.46 μm in 10 Hz-ON group vs. −27.35 ± 13.99 μm in RL group, *p* = 0.04, and vs. −37.46 ± 12.48 μm in 1.2 Hz-ON group, *p* = 0.008; ∆7 days: 14.99 ± 9.54 μm in 10 Hz-ON group vs. −37.96 ± 14.89 μm in RL group, *p* = 0.02, Figure 5A). In contrast, the 1.2 Hz and 10 Hz square wave stimuli had no effect on ChT, neither in the negative lens treated nor the fellow eyes compared to the static and room light groups (Figure 5E). The experiment was repeated, limiting the treatment to 3 days because we wanted to collect samples for gene expression studies comparing the effect of long-term and medium-term treatment. The time course of ChT changes during the first 3 days (Figures 5B,D) was similar to that observed in the long-term treatment experiment (Figures 5A,C). Specifically, there was a rapid decrease in ChT during the first day. ChT changes in the 10 Hz-ON and 10 Hz-OFF flicker groups were not significantly different from that in room light and under static stimuli. However, the chicks treated with 10 Hz square wave stimulus had a significantly thicker choroid than those treated with RL during the first 2 days in LIM eyes (RL vs. 10-square: ∆1 day: −93.80 ± 16.13 μm vs. −30.24 ± 9.85 μm, *p* = 0.01; ∆2 days: −87.58 ± 11.16 μm vs. −34.58 ± 11.48 μm, *p* = 0.049, Figure 5F), and during the first 3 days in the fellow eyes (RL vs. 10-square: ∆1 day: −45.94 ± 12.10 μm vs. 2.72 ± 6.14 μm, *p* = 0.02; ∆2 days: −35.56 ± 12.28 μm vs. 6.07 ± 4.65 μm, *p* = 0.04; ∆3 days: −27.69 ± 12.40 μm vs. 17.89 ± 6.73 μm, *p* = 0.02, Figure 5F). In addition, the ChT of the fellow eyes in 10-square group was also thicker than that in the static stimulus group on the 3rd day (Static vs. 10-square: −30.16 ± 12.62 μm vs. 17.89 ± 6.73 μm, *p* = 0.02, Figure 5F).

We also investigated whether initial changes in ChT that occurred on the first day of lens treatment were correlated with later changes in axial length and vitreous chamber depth. For this analysis, the changes in ChT relative to baseline on day 0 were determined separately for each eye. The values of the contralateral control eyes were then subtracted from those of the lens-treated eyes (ΔX − ΔN). We found that initial changes in ChT relative to baseline between the two eyes (ΔX − ΔN: day 1) were not significantly correlated with changes in axial length and vitreous chamber depth after 7 days of treatment (ΔX − ΔN ChT day 1 vs. ΔX − ΔN AL change day 7: *p* = 0.099, *R*^2^ = 0.0504). In contrast, relative ChT changes at day 5, day 6 and day 7 showed a significant association with myopia development (Supplementary Figure S2A: AL: *R*^2^ = 0.1241, *p* < 0.01; Supplementary Figure S2B: VCD: *R*^2^ = 0.0977, *p* < 0.05). After treatment of 3 days, only axial length changes and ChT showed a weak but significant correlation (medium term treatment: *R*^2^ = 0.0763, *p* < 0.05).

Interestingly, ChT changed significantly during the 7 days treatment in some groups. Pronounced recovery was observed for both 1.2 Hz and 10 Hz OFF stimulation which showed up as an increase in ChT during the last 3 days of the experiment (Supplementary Table S1).

We also investigated whether initial changes in ChT that occurred on the first day of lens treatment were correlated with later changes in choroidal thickness. In an analysis of the entire data set, a significant correlation between changes on day 1 and changes at each subsequent day was observed. Obviously, animals that showed a large decrease in ChT in eyes with negative lenses, compared to their fellow eyes on day 1 also showed a larger difference on subsequent days. However, the significance of the correlation declined steadily over the week of treatment (Supplementary Table S2). In a separate analysis of this relationship in each of the treatment groups, the predictive power was high during the first 3 days in the animals in the static stimulus group and from day 2 to day 6 in the 10 Hz OFF group but there was no correlation with any other stimulus.

### 3.5 Effects of the different visual stimuli on retinal dopamine metabolism

**Figure 6:**
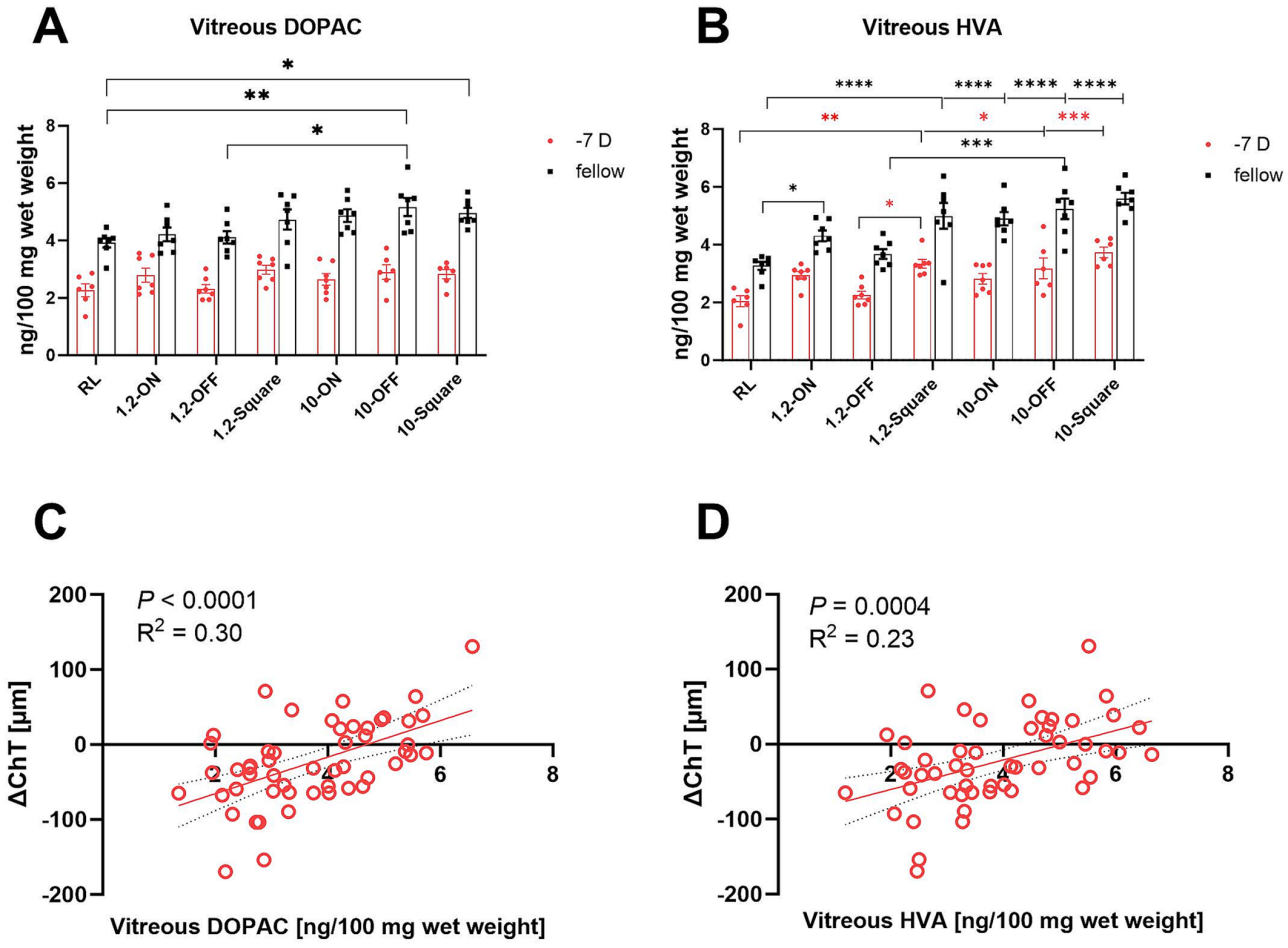
Effect of different stimuli on the level of retinal dopamine metabolites. The 10-Hz OFF and 10-Hz square stimuli increased the level of vitreal DOPAC in the fellow eyes **(A)**. All high-frequency patterns and the 1.2-Hz square stimulus increased the amount of HVA in the vitreal in the fellow eyes. The high frequency stimuli also increased the level of vitreal HVA in LIM eyes, except for the 10-Hz ON stimulus. In addition, the fellow eyes in 1.2-ON group contained more vitreal HVA in comparison to the RL group **(B)**. The change in ChT after 7 days (RL, 10-ON, 10-OFF, 10-square group data are shown) were positively correlated with the level of vitreal DOPAC **(C)** and vitreal HVA **(D)**. Data are shown as mean ± SEM. ^*^p < 0.05, ^**^p < 0.01, ^***^p < 0.001, and ^****^p < 0.0001.

Vitreal levels of dopamine metabolites DOPAC and HVA can be used as a sensitive measure of dopamine release from the retina (33). In line with previous findings (38), negative lens treatment reduced the amount of dopamine metabolites DOPAC and HVA with high significance in all groups (Supplementary Table S3). In addition, frequency (0 Hz, 1.2 Hz, or 10 Hz) and stimuli pattern (ON, OFF, square wave, room light) had a significant main effect on dopamine metabolism (multifactorial ANOVA: DOPAC: treatment and frequency: *p* < 0.0001, Figure 6A; HVA: treatment, frequency and stimuli pattern: *p* < 0.0001, Figure 6B). When vitreal HVA data were grouped according to stimulus type, HVA content was significantly higher when chicks were stimulated with square wave stimuli compared to room light and OFF stimuli, and also higher under ON stimulation than under room light (one-way ANOVA, *p* = 0.0002, Figure 6B). A recent study in our lab (15) found a significant increase in DOPAC and HVA level in chicks kept under 1 Hz-ON vs. 1 Hz-OFF stimulation. Although, there was no significant difference between 1.2 Hz-ON and 1.2 Hz-OFF stimuli in our present study, a similar trend was seen in terms of a higher mean level of vitreal HVA in fellow and −7D lens-treated eyes in the 1.2-ON vs. 1.2 OFF group. When the vitreal HVA data were grouped according to frequency, vitreal HVA was significantly higher under flicker light, compared to room light, and also higher at 10 Hz flicker than at 1.2 Hz flicker (one-way ANOVA, *p* < 0.0001, Figure 6B).

Overall, the largest changes were observed in fellow control eyes under 10 Hz flicker with the OFF stimulus, with significantly higher level of vitreal DOPAC than in room light and 1.2 Hz OFF flicker (10-OFF vs. RL: 5.17 ± 0.31 vs. 3.92 ± 0.17 ng/100 mg wet weight, *p* = 0.003; vs. 1.2-OFF: 4.11 ± 0.21 ng/100 mg wet weight, *p* = 0.02, Figure 6A). Also, the 10 Hz square wave stimulus increased vitreal DOPAC levels in the fellow control eyes. Also, the 1.2 Hz and 10 Hz ON, OFF and square wave stimuli increased the amount of vitreal HVA level in fellow eyes, compared to room light.

In the negative lens-treated eyes, vitreal HVA content was significantly higher in at 10 Hz OFF and 10 Hz square wave stimulation compared to room light (RL vs. 10-OFF: 2.05 ± 0.19 vs. 3.18 ± 0.36 ng/100 mg wet weight, *p* = 0.03; vs. 10-square: 3.73 ± 0.18 ng/100 mg wet weight, *p* = 0.0002, Figure 6B). Vitreal HVA levels were also increased at 1.2 Hz square wave stimulation, compared to room light and to 1.2 Hz OFF stimulation.

After 1 week of treatment, vitreal DOPAC and HVA content were correlated with the changes in ChT (Figures 6C,D) when data from all eyes were pooled. The higher their content, the thicker was the choroid (*p* < 0.0001, *R*^2^ = 0.30; *p* = 0.0004, *R*^2^ = 0.23, respectively, Figures 6C,D).

### 3.6 Effects of ON and OFF stimuli and treatment duration on gene expression

#### 3.6.1 Retina

**Figure 7:**
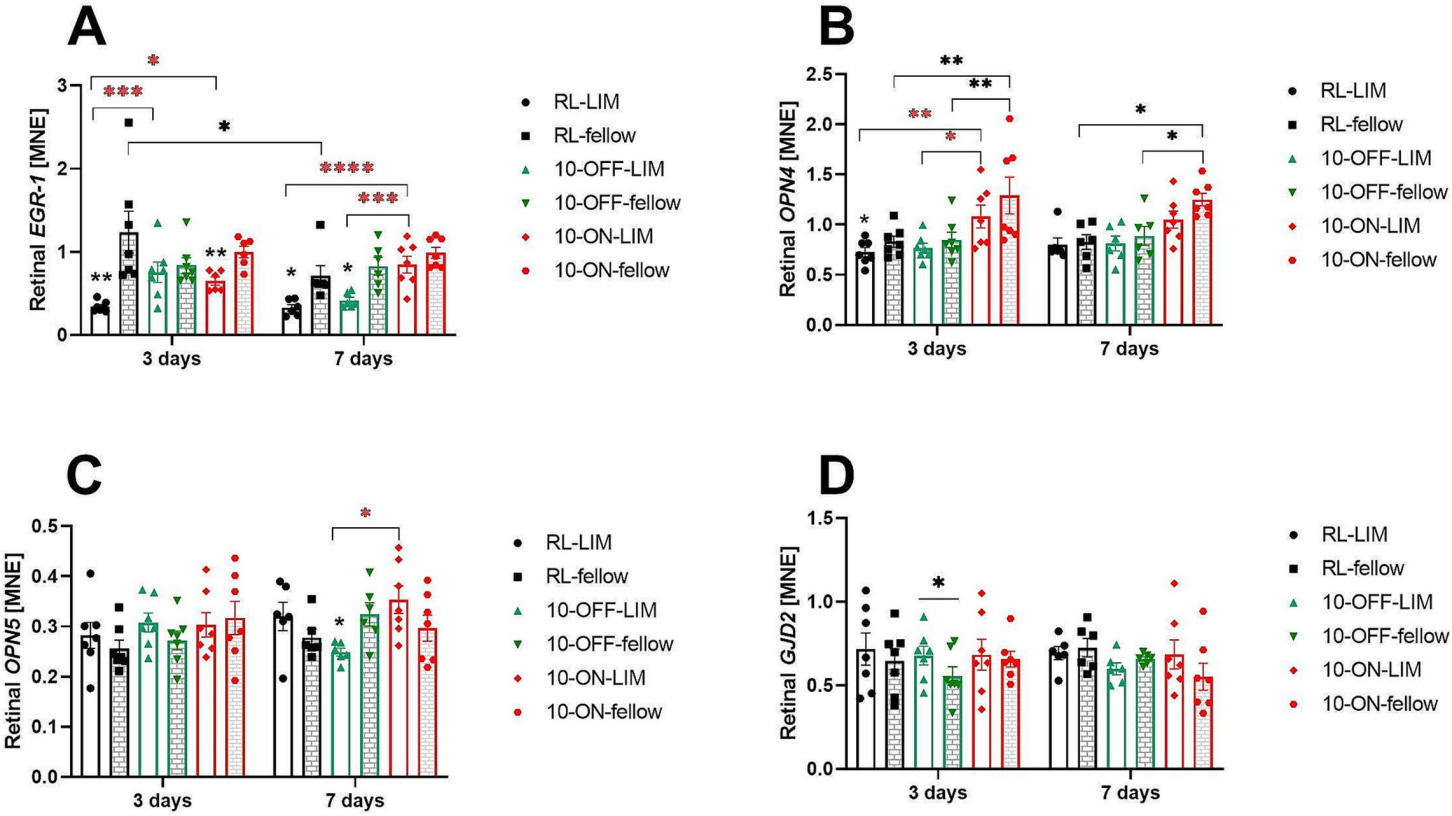
The mRNA expression level of myopia candidate genes in the retina after long or short-term treatment. Negative lens treatment significantly decreased retinal *EGR-1* expression in room light (RL), in 10 Hz ON flicker (after 3 days), and in 10 Hz OFF flicker (after 7 days). Compared to the RL-LIM eyes, *EGR-1* mRNA expression in the lens treated eyes was significantly higher, both in the 10-Hz ON and 10-Hz OFF stimuli, after 3 days. This effect persisted in the 10-ON group in the long-term experiment **(A)**. A temporal effect was observed in the fellow eyes of the RL group, as the level of retinal *EGR-1* mRNA decreased over time **(A)**. After 3 days of treatment, the 10-Hz ON stimulus induced a significant increase in the level of retinal *OPN4* mRNA in both eyes, compared to the RL and 10-OFF groups. However, after 7 days of treatment, this effect was observed only in the fellow eyes **(B)**. The expression of retinal *OPN5* mRNA was higher in LIM eyes in the 10-ON group than that in the 10-OFF group **(C)**. In the short-term experiment, only the 10-OFF group showed an interocular effect in the expression of retinal *GJD2* mRNA, which was an elevation in the LIM eyes **(D)**. Data were shown as mean ± SEM. ^*^p < 0.05, ^**^p < 0.01, ^***^p < 0.001, and ^****^p < 0.0001.

Expression of candidate genes that were previously associated with myopia development was measured after 3 and 7 days of negative lens treatment, both in room light as well as with 10-Hz ON and 10-Hz OFF flicker light (Figure 7). As found in previous studies (34), *EGR-1* mRNA levels decreased in negative lens-treated eyes. This effect was found in room light, both after 3 days (RL-LIM vs. RL-fellow: 0.34 ± 0.02 vs. 1.24 ± 0.25, *p* = 0.01) and 7 days (RL-LIM vs. RL-fellow: 0.33 ± 0.04 vs. 0.71 ± 0.12, *p* = 0.02, Figure 7A). *EGR-1* mRNA expression was also lower in the negative lens-treated eyes compared to fellow eyes at 10 Hz ON flicker after 3 days of treatment (0.65 ± 0.05 vs. 1.00 ± 0.07, *p* = 0.047) and 10 Hz OFF flicker after 7 days (0.42 ± 0.04 vs. 0.83 ± 0.11, *p* = 0.02). However, *EGR-1* mRNA expression in eyes with negative lenses was still significantly higher at 10 Hz ON (0.65 ± 0.05, *p* = 0.02) and OFF stimulation after 3 days of treatment (0.76 ± 0.12, *p* = 0.0009), compared to room lighting (0.34 ± 0.02). The effect persisted after 7 days with 10 Hz ON stimulation (0.85 ± 0.11, *p* < 0.0001 to RL, *p* = 0.0008 compared to 10 Hz OFF stimulation).

We also found a small reduction in the level of retinal *OPN4* mRNA in eyes with negative lenses after 3 days under room lighting (RL-LIM vs. fellow: 0.73 ± 0.05 vs. 0.83 ± 0.05, *p* = 0.03, Figure 7B). The perhaps most significant finding was that 10 Hz ON exposure stimulated the expression of *OPN4* mRNA in both eyes after 3 days and in fellow eyes after 7 days (Figure 7B). There was no change in the level of retinal *OPN5* mRNA after 3 days of treatment. After 1 week of treatment, retinal *OPN5* mRNA expression was higher in the negative lens-treated eyes after 10 Hz ON stimulation (0.35 ± 0.03) compared to 10 OFF stimulation (0.25 ± 0.01, *p* = 0.01, Figure 7C).

A small increase in retinal *GJD2* mRNA was observed in the eyes treated with negative lenses under 10 Hz OFF stimulation after 3 days, compared to fellow eyes (0.68 ± 0.06 vs. 0.55 ± 0.06, *p* = 0.047, Figure 7D).

After 3 day of treatment, changes in ChT and retinal *EGR-1* mRNA levels were positively correlated, showing that retinal *EGR-1* mRNA levels increase as the choroid thickened. This correlation was significant (*p* = 0.007), although only 17% of the change in ChT was explained by changes in *EGR-1* in mRNA (*R*^2^ = 0.17). No such correlation was found after 7 days of treatment, nor for any of the other studied genes.

#### 3.6.2 Choroid

**Figure 8:**
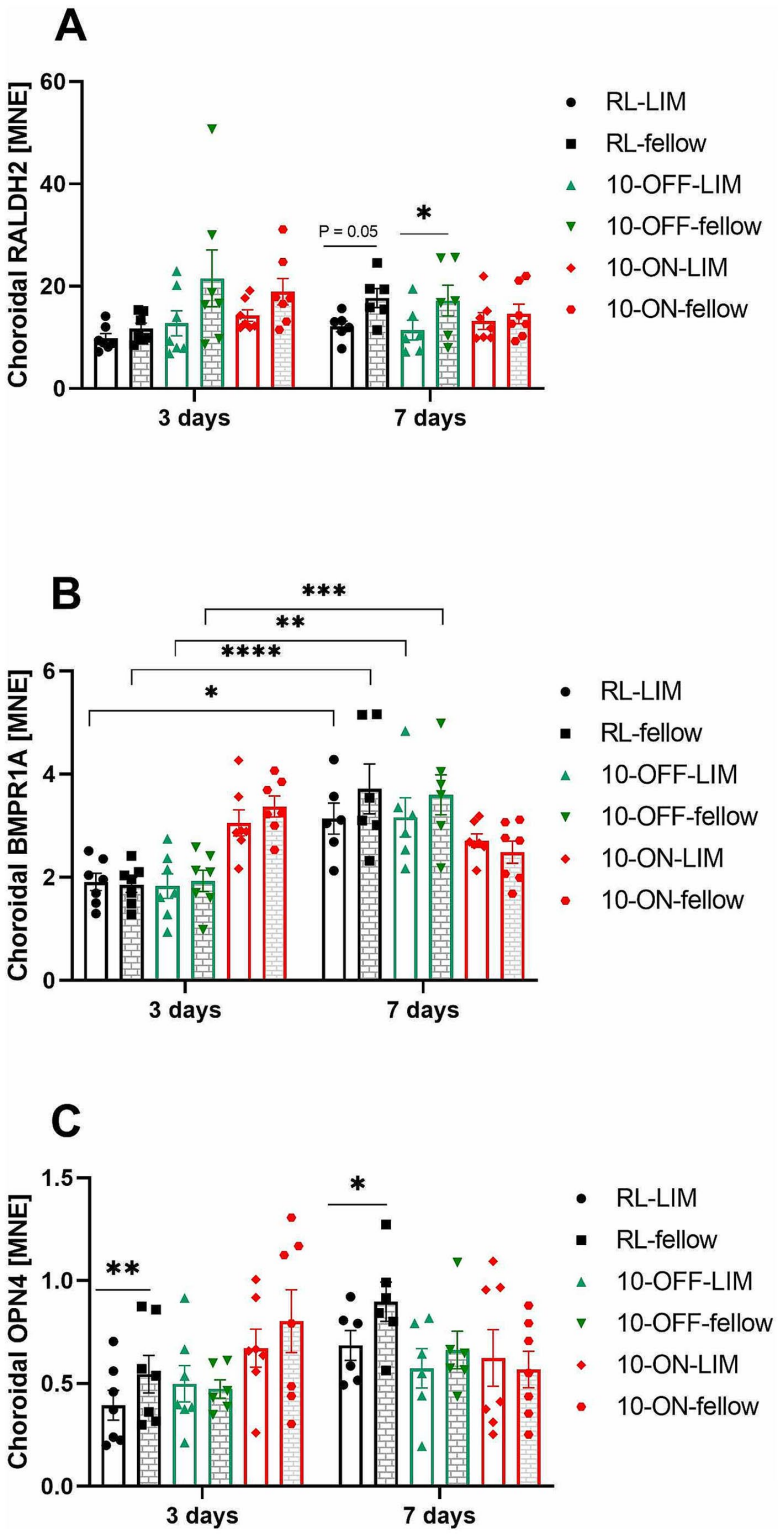
The mRNA expression level of myopia-related genes in the choroid after long or short-term treatment. The expression of choroidal *RALDH2* mRNA was decreased by −7D lens in the RL and 10-OFF group after treatment for 7 days **(A)**. In both eyes of the RL and 10-OFF groups, a significant temporal effect was shown in the expression of choroidal *BMPR1A* mRNA, which was elevated over time **(B)**. The expression of choroidal *OPN4* mRNA was found to be decreased in LIM eyes in the RL group **(C)**. Data were shown as mean ± SEM. ^*^p < 0.05, ^**^p < 0.01, ^***^p < 0.001, and ^****^p < 0.0001.

There was no significant change in choroidal *RALDH2* mRNA during 3 days of treatment. However, the level of *RALDH2* expression was significantly lower in the lens treated eyes compared to the fellow eyes with 10 Hz OFF stimulation after 7 days (11.44 ± 1.92 vs. 17.23 ± 3.01, *p* = 0.03, Figure 8A). A similar trend was observed in room lighting (12.18 ± 1.06 vs. 17.72 ± 1.83, *p* = 0.05). After 7 days, also choroidal *BMPR1A* mRNA changed in both eyes, both in room lighting and 10 Hz OFF stimulation (RL-LIM: 3.14 ± 0.30; RL-fellow: 3.72 ± 0.48; 10-OFF-LIM: 3.16 ± 0.38; 10-OFF-fellow: 3.60 ± 0.39). At 7 days, there was a higher level of *BMPR1A* expression compared to 3 days (RL-LIM: 1.91 ± 0.17, *p* = 0.01; RL-fellow: 1.86 ± 0.15, *p* < 0.0001; 10-OFF-LIM: 1.83 ± 0.24, *p* = 0.005; 10-OFF-fellow: 1.93 ± 0.21, *p* = 0.0002, Figure 8B). No temporal effect was found in 10-ON group. An interocular difference was observed in the expression of choroidal *OPN4* mRNA during both the early (LIM vs. fellow: 0.39 ± 0.07 vs. 0.55 ± 0.09, *p* = 0.003) and late periods (LIM vs. fellow: 0.69 ± 0.07 vs. 0.90 ± 0.10, *p* = 0.04, Figure 8C) of treatment in the RL group. This difference was not observed in the other groups.

After treatment for 3 days, ChT change and choroidal *RALDH2* mRNA level were found to be positively correlated. This correlation was significant (*p* = 0.045), although only 10% of the change in ChT can possibly be explained by changes in the amount of choroidal *RALDH2* mRNA expression. No significant correlation was found for the other genes.

## 4 Discussion

We have investigated how different computer-generated dynamic visual stimuli, assumed to predominantly stimulate retinal ON and OFF pathways, in combination with or without imposed optical defocus, affect ChT thickness, axial length, refraction and the expression of some biomarkers of myopia, including dopamine. The time kinetics was also studied, by measuring after 3 h, and 3 and 7 days of continued treatment. The potential predictive power of these variables for future refractive development was statistically analyzed.

### 4.1 Temporal and spatial frequency during ON or OFF stimulation determine changes in ChT

After short-term treatment of 3 h, the effect of ON/OFF stimuli on ChT varied with the field sizes of the dynamic checkerboard patterns and their temporal frequency. Both dynamic ON and OFF stimuli caused choroidal thinning in chicks with normal vision, with the largest effect at the smallest field size used. Only one type of stimulus, a 1.2 Hz pattern cycle checkerboard with medium field sizes of about 2.9 deg. visual angle, led to thickening of the choroid. It is well known that stimulus size plays an important role in the ON/OFF response of retinal ganglion cells (RGCs). With an increase of stimulus size, the amplitudes of both, ON- and OFF-center RGCs, decrease. When a full-field stimulus was presented to mice *in vivo*, approximately half of the OFF-center RGCs altered their sign into either OFF/ON or ON only. The sign switch was derived from the area surround the receptive field (39). In humans, ON and OFF responses in the primary visual cortex have different spatial characteristics: large gratings with low spatial frequencies activate more OFF-dominated neurons, while narrow gratings with high spatial frequencies shifts the responses to ON-dominated neurons (40). ON and OFF ganglion cells exhibit an asymmetrical pattern in their temporal adaptation to photopic and scotopic conditions. Specifically, under photopic conditions, both ON and OFF ganglion cells demonstrate similar temporal characteristics. However, under scotopic conditions, ON cells shift their tuning towards low temporal frequencies, whereas OFF cells respond to higher frequencies (41). In mice, the response of both ON and OFF retinal ganglion cells increased with temporal frequencies ranging from 0.15 to 3 Hz. Beyond 3 Hz, their responses began to decrease (41). In our study, chickens were stimulated under photopic conditions, with an ambient illumainance of 400 lux. Assuming that findings in mice and humans can be extrapolated to birds, a smaller stimulus size combined with relatively low stimulation frequency should induce a stronger response in both ON and OFF cells. In fact, differences between ON and OFF stimulation were only observed with medium-sized fields in the checkerboard at a frequency of 1.2 Hz. In this case, ON stimuli caused thickening of the choroid, and OFF stimuli resulted in choroidal thinning. One reason for the generally not consistent differences between ON versus OFF stimulation in the current study is that the checkerboard fields may have been too large to stimulate antagonistic receptive fields. Diedrich and Schaeffel (42) used multi-electrode recordings in the chick retina and found a visual acuity of the chicks of about 5 cyc/deg. and a rather low maximal contrast sensitivity of about 10 at 1.7 cyc/deg. (10% contrast is detected). The strongest responses of ganglion cells were measured with stimuli of 1.64 cyc/deg. In the current study, we have a minimal 1.7 deg. visual angle (SQS), equivalent to about max 0.3 cyc/deg. It is not known, whether the antagonistic ON/OFF receptive fields are activated at this field size. If not, only temporal ON/OFF signals will be generated.

In young human subjects, a computer-generated dynamic ON stimulus at 1 Hz, similar to the one in the current study increased ChT while the OFF version of it decreased it (15). The authors of this study translated the ON and OFF signals into practical applications for humans: reading white text on a black background stimulates the ON pathway, while reading black text on a white background stimulates the OFF pathway (43). The magnitudes of these effects of contrast polarity on ChT were greater than that of dynamic ON/OFF square stimulations in humans and chicks. This difference in magnitude could be attributed to the significantly smaller size of the stimuli in the former case, as speculated by the authors. Nevertheless, we observed in this study that a medium size ON stimulus was in fact more effective compared to the small size, which was used in the previous study (15), in chickens. The reason for this difference in results between the two studies remains unclear, as the same stimuli were used. The only known difference between the two studies that we are aware of, is the different White Leghorn hybrid line used. Furthermore, our findings revealed that only the OFF stimulus with a frequency of 10 Hz had an enhancing effect on ChT in LIM eyes. The reason could be that the OFF RGCs activate preferential over ON RGCs in a frequency band around 10 Hz, as predicted in a model (44).

The effects of the ON and OFF stimuli on refractive error development also vary. Both the 1-Hz ON and OFF stimuli of small squares promoted the development of LIM in chickens (15). In another study, a 4-Hz OFF stimulus was observed to diminish positive lens-induced hyperopia, while a 4-Hz ON stimulus mitigated the development of LIM in chickens (45). Likewise, blocking the ON response inhibited the progression of LIM, whereas blocking the OFF response prevented the development of positive lens-induced hyperopia (46). In the human visual cortex, ON cortical pathways exhibit higher contrast sensitivity compared to OFF cortical pathways, with this discrepancy escalating with luminance range. The asymmetrical characteristics present in ON and OFF responses add complexity to the assessment of effects, particularly when considering tuning parameters. However, in cases of myopia, where the eye elongates, the ON pathways become less responsive and less sensitive (47).

### 4.2 Differential effect of high flicker frequency on the initial choroidal response to positive and negative lens treatment

Short term treatment with negative lenses induced a significantly larger response (reduction in choroidal thickness) in the negative lens-treated eyes with 10 Hz ON flicker compared to 10 Hz OFF flicker, whereas 3 h of plus lens treatment induced a larger response (increase in ChT) with stimulation of the ON system. It was already discussed since long that different retinal circuits with different temporal characteristics are involved in the processing of hyperopic and myopic defocus (48), which might than lead to the differential effect of high frequency ON and OFF flicker on the size of the choroidal response as shown in our study. In addition, it was recently discussed that “choroidal thickening induced by positive defocus may be triggered by metabolic constraints, rather than representing a “third mechanism of focusing the eye” as first proposed by Wallman et al. (1, 49).

### 4.3 The initial extent of ChT thinning (after 1 day) does not predict the amount of negative lens-induced myopia

ChT is inversely correlated with axial length in myopia (50), as well as with refractive error and visual acuity in high myopes (51, 52). In addition, subfoveal ChT in humans is an independent predictor for myopic maculopathy progression (53). In a large cohort and longitudinal study performed in young adults, the authors found that each 10 μm increase in baseline ChT was associated with a refractive change of 0.006 D/year and of 0.003 mm/year in axial length (54). In addition to forecasting the natural eye growth process, ChT can also be used to predict or assess the effectiveness of myopia control methods, such as repeated low-level red-light therapy (55) and low-concentration atropine (56). Animal experiments have shown that baseline ChT is correlated with baseline refraction and deprivation-induced myopia development in pigmented guinea pigs, but not in the albino ones (57). On the other hand, it has been shown that ChT as an independent parameter cannot predict visual acuity in high myopes after adjustment for spherical equivalent and the presence of pathological myopic lesions (58). Similar evidence has been found in chickens, indicating that baseline ChT is unable to predict the extent of the final myopic shift in form-deprived chickens (7). However, it has been shown to be predictive of ocular growth in chickens with normal vision (6), where a thicker choroid is associated with reduced eye growth. However, this relationship is not as quantitatively precise as the correlation between refractive error and axial length.

A number of studies have shown an association between a treatment-induced reduction in eye length growth and thickening of the choroid. As for example a brief period of normal vision during negative lens-wearing effectively slow down myopia development, inducing a choroid thickening of 91 μm and less axial elongation of 183 μm (59). In addition, the dopamine receptor agonist apomorphine reduces axial elongation by 184 μm and increases choroidal thickness by 42 μm in LIM chickens (60). This may also explain why 0.01% atropine is not effective in slowing myopia progression in children in the long term, as there was no significant increase in ChT after 6 months of use (61).

In our study, the choroidal thickness decreased significantly less in the 10-OFF group. This effect was already seen after 3 h of treatment. The 10 Hz OFF treated chicks also had a thicker choroid after 7 days, but there was no significant difference in the lens-induced myopic shift in this group compared to the control group. It seems that less ChT reduction does not mean less eye growth. This result therefore supports earlier findings of our groups and others that a decrease in choroidal thickness induced by artificial stimuli has no predictive power for the change in eye length growth. With the exception of one stimulus, all artificial stimuli tested in this study led to choroidal thinning. ChT decreased strongly during the first day of stimulation followed by a partial or full recovery at day 7. Initial choroidal thinning (1 day) induced by negative lens wear under a number of artificial stimuli did not predict the amount of myopia induced by negative lenses after 7 days, while ChT changes that occurred later during the treatment period showed a significant association. One has to keep in mind that the extent to which the choroid can thin has a natural lower limit, which potentially had an impact on the results of the correlation analysis. Also, the result of the present study does not provide much information about the predictive power of initial choroidal thickening, since only one stimulus induced this effect (1.2 Hz-ON after 3 h of treatment). A current review article by Ostrin et al. (62) states that “current evidence is not sufficient to speculate that short-term choroidal thickening can be used as a biomarker of treatment efficacy for myopia control therapies.” Therefore, future studies should focus on stimuli that thicken the choroid in order to analyze the predictive power of choroidal thickening.

### 4.4 Influence of artificial stimuli and lens treatment on dopamine metabolism and the correlation with ChT

As expected, negative lens treatment reduced dopamine release as measured by changes in the amount of dopamine metabolites in the vitreous. On the other hand, dopamine release was strongly stimulated by high frequency flicker and also-although to a lesser extent- by low frequency flicker; both in fellow control eyes (DOPAC and HVA) and in the lens-treated eyes (HVA). This result thereby supports previous findings in chicks showing that frequencies around 10 Hz (stroboscopic light (63)) optimally activate dopaminergic amacrine cells and therefore stimulate retinal dopamine release. In addition, a study in guinea pigs demonstrated that 0.5 Hz luminance flicker increase the levels of the dopamine metabolites (64). Interestingly, HVA level was highest in the group of chicks kept under square wave stimulation. However, as HVA levels were equally elevated in both control and lens-treated eyes, the lens-induced effect persisted in all treatment groups.

The amount of dopamine release was positively correlated with choroidal thickness. This result thereby confirms an association between both parameters that was also recently described and discussed in a study by Mathis et al. (65). As no dopamine receptors have been identified in the choroid, it was supposed that a direct effect of the transmitter in this tissue is unlikely (66). However, Mathis et al. showed that retinal and choroidal dopamine levels are correlated, suggesting a role for dopamine in choroidal thickness changes. Further studies are needed to clarify the exact nature of the pathway linking retinal dopamine release to ChT changes.

### 4.5 Influence of artificial stimuli and lens treatment on candidate gene expression changes and their correlation with changes in ChT

Changes in visual input and signals within the retina can lead to changes in ChT, scleral structure and consequently axial elongation of the eye. With the use of large-scale screening technologies, a number of molecules have been found to be associated with the development or prevention of induced myopia and therefore postulated to play a role in the regulation of ocular growth (67).

Most consistent across studies and animal models were changes in the expression of the immediate early gene *EGR-1* in correlation with induced defocus and deprivation. *EGR-1* knockout mice exhibit axial myopia, highlighting its essential role in eye growth (68). The expression pattern of *EGR-1* aligns with the trajectory of eye growth: it is downregulated in eyes developing LIM (lens-induced myopia) and upregulated during recovery from LIM (69). In our study, we confirmed that lens treatment reduced the expression of *EGR-1* under room light conditions. Interestingly, we found a significant positive correlation of ChT changes and retinal *EGR-1* mRNA levels after 3 days of treatment, i.e., a low retinal *EGR-1* level was correlated with a thinner choroid. However, whether a causal relationship exists requires further investigation, also because there was no significant correlation between both parameters after 1 week and the extent of the correlation was relatively low (only 17% of the change in ChT could be explained by changes in *EGR-1* expression). Possibly, ChT changes might be regulated at least partly by visual signals derived from the retina, including *EGR-1*. However, after longer treatment, a disassociation takes place. This is also reflected in the increased mRNA expression of *EGR-1* in the LIM retina of the 10-ON groups which does not correspond to a reduced eye growth after 1 week, indicating that *EGR-1* expression may not/not any more correlate with the rate of eye growth after long treatment periods.

The expression of the gap junction protein 2, also known as connexin-36, was found to decrease in the retinas of guinea pigs with FDM (19) and LIM (70) whereas functional connexin-35 increased in the cone-dominated myopic chicken retina (71). In mice, no change in *GJD2* mRNA was found but an increased phosphorylation of connexin-36 which could indicate increased functional gap junction coupling of AII amacrine cells in the rod-dominated myopic mouse retina. This might be a possible adaptation to adjust to the altered noisy signaling status (72). AII amacrine cells (ACs), coupled by connexin-36, segregate signals into ON and OFF pathways. In our study, only the retinas of 10 Hz OFF stimulated chicks showed a higher level of *GJD2* mRNA compared to the fellow eyes after 3 days of treatment. This effect in gene expression in 10 Hz-OFF was not correlated with differential changes in ChT in this treatment group at that time point. In addition, the increase in *GJD2* mRNA expression level was only temporal and not seen after 1 week of treatment.

Violet light has been found to provide protective effects against lens-induced myopia (LIM) and to cause choroidal thickening in mice. These effects are dependent on the function of retinal *OPN5* (73). Violet light exposure also upregulated *EGR-1* expression in the chicken chorio-retinal tissues (74), an effect subsequently confirmed in mice to be mediated by *OPN*5 (75). In our study, we observed significantly higher levels of *EGR-1* and *OPN5* mRNA expression in the LIM eyes of the 10-ON group compared to the 10-OFF group after 1 week of treatment. Given that our light source did not emit violet light, it seems plausible that the alteration in *OPN5* and *EGR-1* expression are independent of each other in this particular case.

The blue light sensitive non-visual opsin melanopsin (*OPN4*) is expressed in the choroid and in intrinsically photosensitive ganglion cells (ipRGCs) in the retina which, like rods and cones, provide essential input to dopaminergic amacrine cells (76). *OPN4* knock-out mice exhibit greater hyperopia compared to wildtype mice; however, they are also more susceptible to form-deprivation myopia (FDM) (20). We did not detect any lens-induced changes in retinal *OPN4* expression after one week, but its level was significantly elevated in the 10 Hz ON group compared to the 10 Hz OFF group, in fellow and lens treated eyes after 3 days of treatment. Since short-term experiments showed a significant thinner choroid during short-term 10 Hz ON stimulation (Figure 8B), a higher retinal *OPN4* level might be associated with a thinner choroid, which fits to the *OPN4* knock-out results. It was also observed in *OPN4* knock-out mice that the choroidal thickening in response to the light stimulation was absent (77). In contrast to extensive knowledge about the function of melanopsin in the retina, its role in the choroid is currently unknown. Interestingly, it was recently concluded that melanopsin may regulate diurnal or defocus-induced changes in ChT, “including the intriguing possibility that the choroid may be photosensitive” (78). Indeed, the opsin genes including *OPN4* were found to be expressed in the chick choroid (79). We observed that the *OPN4* mRNA expression was decreased in the LIM eyes compared to the fellow eyes in the RL group, both in short-term and long-term experiment. The expression of *OPN4* in the choroid might therefore play a role in the thickness response.

A significant down-regulation of the bone morphogenetic protein receptor *BMPR1A* was observed in the choroid of chickens after 48 h of treatment with a +10D lens, while no such effect was observed in the retina (24). Interestingly, our study identified a temporal effect on *BMPR1A* mRNA expression. Specifically, prolonged treatment resulted in elevated mRNA levels in both eyes of the RL and 10-OFF groups. In contrast, the 10-ON group exhibited an opposite trend in mRNA expression. The temporal upregulation of BMP is necessary for early ocular lens development in chickens (80). Therefore, the temporal elevation might be related with eye development as well.

## 5 Summary

In conclusion, our study confirms that negative lens treatment thins the choroid in chickens, and that the effect can be modulated by artificial visual stimuli in their environment. However, the induced increase in ChT with 1.2 Hz ON flicker was too small to generate long-term changes in myopia development. We found that the amplitude of choroidal thinning on day 1 of lens treatment had no predictive value for the amount of myopia that developed in 7 days, whereas relative changes in ChT were predictive from day 5 on. Parameters of the used ON/OFF stimuli tuning, such as field sizes of the checkerboard and cycle frequency induce varying effects on ChT, highlighting the need for careful analysis of these parameters to achieve inhibition of myopia.

## Data Availability

The original contributions presented in the study are publicly available. This data can be found here: https://www.ncbi.nlm.nih.gov/nuccore/XM_046919995.1.

## Footnotes

^1^ https://imagej.net/software/fiji/

## Supplementary material

SUPPLEMENTARY FIGURE 1 Light spectrum perceived by the chicks in the experimental set up “arena”.

SUPPLEMENTARY FIGURE 2 Significant correlation of relative changes in choroidal thickness with axial length **(A)** and vitreous chamber depth **(B)** after 7 days of treatment. Interocular differences between the two eyes are shown (X = lens treated eye; N = normal fellow eye).
