# Supplementary figures and images for: Effects of computer-generated patterns with different temporal and spatial frequencies on choroidal thickness, retinal dopamine and candidate genes in chickens wearing lenses
Source: Front Med (Lausanne). 2024 Dec 10;11:1469275. doi: 10.3389/fmed.2024.1469275 (PMC11666368; doi:10.3389/fmed.2024.1469275)

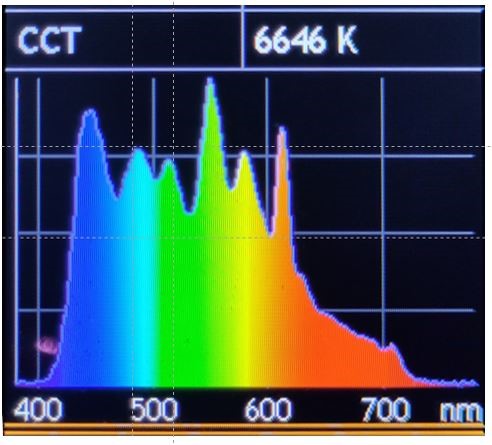

Supplement: SUPPLEMENTARY FIGURE 1 — Light spectrum perceived by the chicks in the experimental set up “arena”. [file Image_1.JPEG]

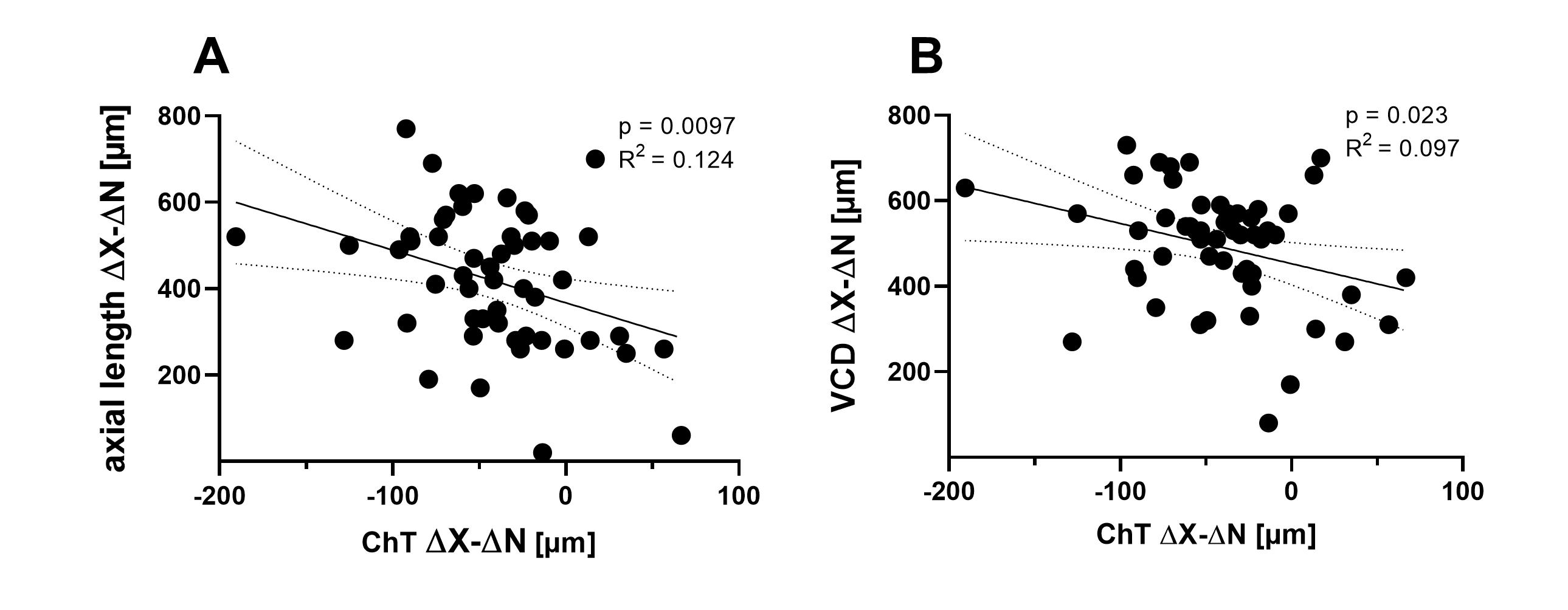

Supplement: SUPPLEMENTARY FIGURE 2 — Significant correlation of relative changes in choroidal thickness with axial length (A) and vitreous chamber depth (B) after 7 days of treatment. Interocular differences between the two eyes are shown (X = lens treated eye; N = normal fellow eye). [file Image_2.JPEG]
