# Supplementary material for: Effects of computer-generated patterns with different temporal and spatial frequencies on choroidal thickness, retinal dopamine and candidate genes in chickens wearing lenses
Source: Front Med (Lausanne). 2024 Dec 10;11:1469275. doi: 10.3389/fmed.2024.1469275 (PMC11666368; doi:10.3389/fmed.2024.1469275)
Supplement: Supplementary file 3 [file Table_1.DOCX]

**Table 1.** Significant temporal changes in ChT compared to the change on the first day.

| Comparisons | Eyes | 95% CI of difference | P value |
| --- | --- | --- | --- |
| ∆1 day vs. ∆2 days | None | None | None |
| ∆1 day vs. ∆3 days | 1.2-OFF-fellow | -69.62 to -4.777 | 0.02 (*) |
| ∆1 day vs. ∆4 days | 1.2-OFF-fellow  10-ON-fellow  1.2-Square-LIM | -81.23 to -16.39  -66.15 to -9.763  -68.64 to -10.75 | 0.0008 (***)  0.003 (**)  0.003 (**) |
| ∆1 day vs. ∆5 days | 1.2-OFF-LIM  1.2-OFF-fellow  10-OFF-LIM  10-OFF-fellow  10-ON-fellow  1.2-Square-LIM | -72.94 to -8.101  -85.49 to -20.65  -75.83 to -10.98  -80.26 to -15.42  -69.92 to -13.53  -76.60 to -18.71 | 0.008 (**)  0.0002 (***)  0.004 (**)  0.001 (***)  0.0009 (***)  0.0002 (***) |
| ∆1 day vs. ∆6 days | 1.2-OFF-LIM  1.2-OFF-fellow  10-OFF-LIM  10-OFF-fellow  10-ON-fellow  1.2-Square-LIM  10-Square-fellow | -87.44 to -22.60  -87.27 to -22.43  -90.49 to -25.65  -87.80 to -22.96  -95.36 to -38.97  -76.46 to -18.57  -59.94 to -2.050 | 0.0001 (***)  0.0001 (***)  <0.0001 (****)  <0.0001 (****)  <0.0001 (****)  0.0002 (***)  0.03 (*) |
| ∆1 day vs. ∆7 days | 1.2-OFF-LIM  1.2-OFF-fellow  10-OFF-LIM  10-OFF-fellow  10-ON-fellow  1.2-Square-LIM | -85.82 to -20.98  -89.48 to -24.64  -93.77 to -28.92  -90.36 to -25.52  -90.98 to -34.59  -85.81 to -27.92 | 0.0002 (***)  <0.0001 (****)  <0.0001 (****)  <0.0001 (****)  <0.0001 (****)  <0.0001 (****) |
