# Supplementary material for: Effects of computer-generated patterns with different temporal and spatial frequencies on choroidal thickness, retinal dopamine and candidate genes in chickens wearing lenses
Source: Front Med (Lausanne). 2024 Dec 10;11:1469275. doi: 10.3389/fmed.2024.1469275 (PMC11666368; doi:10.3389/fmed.2024.1469275)
Supplement: Supplementary file 4 [file Table_2.DOCX]

**Table 2.** Significant temporal changes in ChT compared to the change on the first day (X-N)

| Comparisons | Eyes | R^2^ | P value |
| --- | --- | --- | --- |
| ∆1 day vs. ∆2 days | all groups  Static  1.2-Square | 0.236  0.796  0.539 | 0.0001 (***)  0.007 (**)  0.04 (*) |
| ∆1 day vs. ∆3 days | All groups  Static  10-OFF | 0.132  0.696  0.628 | 0.006 (**7)  0.02 (*)  0.03 (*) |
| ∆1 day vs. ∆4 days | All groups  10-OFF | 0.119  0.715 | 0.009 (**)  0.02 (*) |
| ∆1 day vs. ∆5 days | All groups  10-OFF | 0.163  0.837 | 0.002 (**)  0.004 (**) |
| ∆1 day vs. ∆6 days | All groups  10-OFF | 0.082  0.795 | 0.04 (*)  0.007 (**) |
| ∆1 day vs. ∆7 days | All groups | 0.111 | 0.014 (*) |
