# Supplementary material for: Effects of computer-generated patterns with different temporal and spatial frequencies on choroidal thickness, retinal dopamine and candidate genes in chickens wearing lenses
Source: Front Med (Lausanne). 2024 Dec 10;11:1469275. doi: 10.3389/fmed.2024.1469275 (PMC11666368; doi:10.3389/fmed.2024.1469275)
Supplement: Supplementary file 5 [file Table_3.DOCX]

**Table 3.** The concentration of retinal DA metabolites and the interocular comparisons by paired t-test.

| Metabolite | Group | LIM eyes | Fellow eyes | P value |
| --- | --- | --- | --- | --- |
| Vitreous DOPAC  (ng/100 mg wet weight) | RL | 2.27 ± 0.22 | 3.92 ± 0.17 | < 0.0001 |
|  | 1.2-ON | 2.79 ± 0.24 | 4.22 ± 0.24 | < 0.0001 |
|  | 1.2-OFF | 2.32 ± 0.15 | 4.11 ± 0.21 | < 0.0001 |
|  | 1.2-Square | 2.98 ± 0.16 | 4.73 ± 0.35 | < 0.0001 |
|  | 10-ON | 2.65 ± 0.20 | 4.87 ± 0.22 | < 0.0001 |
|  | 10-OFF | 2.91 ± 0.25 | 5.17 ± 0.31 | < 0.0001 |
|  | 10-Square | 2.83 ± 0.16 | 4.96 ± 0.18 | < 0.0001 |
| Vitreous HVA  (ng/100 mg wet weight) | RL | 2.05 ± 0.19 | 3.27 ± 0.14 | 0.0003 |
|  | 1.2-ON | 2.94 ± 0.13 | 4.31 ± 0.19 | < 0.0001 |
|  | 1.2-OFF | 2.26 ± 0.13 | 3.68 ± 0.17 | < 0.0001 |
|  | 1.2-Square | 3.34 ± 0.15 | 5.00 ± 0.44 | < 0.0001 |
|  | 10-ON | 2.82 ± 0.18 | 4.90 ± 0.23 | < 0.0001 |
|  | 10-OFF | 3.18 ± 0.36 | 5.24 ± 0.35 | < 0.0001 |
|  | 10-Square | 3.73 ± 0.18 | 5.60 ± 0.20 | < 0.0001 |
